# A computational analysis of Telegram’s narrative affordances

**DOI:** 10.1371/journal.pone.0293508

**Published:** 2023-11-14

**Authors:** Tom Willaert

**Affiliations:** Brussels School of Governance, Vrije Universiteit Brussel, Brussels, Belgium; UTM Skudai: Universiti Teknologi Malaysia, MALAYSIA

## Abstract

This paper offers an empirical investigation of the narrative profiles afforded by public, one-way messaging channels on Telegram. We define these narrative profiles in terms of the contribution of messages to a thread of narrative continuity, and test the double hypothesis that 1) Telegram channels afford diverse narrative profiles, corresponding with distinct vernacular uses of the platform’s features, and that 2) networks of Telegram channels sampled from thematically distinct seed channels lean towards distinct profiles. To this end, we analyse the textual contents of 2,724,187 messages from 492 public messaging channels spanning five thematic networks. Our computational method builds up the narrative profiles by scrolling down channels and classifying each message according to its narrative fit with the surrounding messages. We thus find that Telegram channels afford several distinct storytelling profiles, which tend to defy traditional notions of narrative coherence. We furthermore observe correspondences between the thematic orientations of channels and their narrative profiles, with a preference for disparate profiles in channels pertaining to conspiracy theories and far-right counterculture, a preference for coherent profiles in channels pertaining to cryptocurrencies, and mixed types in channels pertaining to disinformation about the war in Ukraine. These empirical observations thus inform our further theorization on how platform features allow users to construct and shape narratives online.

## Introduction

Contemporary scholarship paints a dystopian picture of social media as drivers of societal fragmentation and polarization [1]. Platforms including Facebook, YouTube, Reddit, Twitter (presently named “X”), Instagram, 4chan, and TikTok have thereby become associated with the spread of misinformation, conspiracy theories, and other adverse phenomena with strong narrative foundations [2]. The messaging platform Telegram in particular has been shown to attract extreme actors “deplatformed” from other social media [3], and to propagate misinformation, hate, and anti-establishment narratives through a rapidly expanding public network of interconnected, one-way messaging channels [4–6]. Yet for all efforts in documenting the proliferation of antagonistic narratives online, much still remains to be understood about the role of social media in bringing about such potentially harmful stories [7]. How, fundamentally, does a platform like Telegram, through its technical features, allow users to construct and shape narratives [8, p. 515]?

The objective of this study is to address this question empirically, and to describe links between Telegram’s platform features and its modes of storytelling, starting from textual trace data mined from the platform [9]. Investigating this interface between platform features and narrative requires conceptual and methodological advances in the analysis of social media texts. This most notably concerns the elaboration of machine-guided reading methods that operationalize the concepts of “narrative” and “narrative affordances” so that these can be quantified and extracted from texts. As will follow from this introduction, we argue that such methods can be developed by integrating elements from 1) “distant reading” approaches to digital texts that define narratives in terms of networks of “actants”, and 2) “close reading” approaches that conceptualize the specific narrative affordances of social media’s technical features. After laying this conceptual groundwork, we proceed to identify the aspects of Telegram that will form the empirical object of this study, and outline our hypotheses.

### Conceptual foundations: Actantial networks and narrative progression

As part of an emerging paradigm of computational approaches to humanities scholarship, typically referred to as “distant reading” [10, 11], the extraction of narratives from large collections of digital texts has developed into an active area of research. In this field, structuralist theories of narrative have proven to lend themselves well to computational operationalizations [12, 13], in particular Greimas’s actantial schema [14, 15]. This schema conceptualizes narratives in terms of relationships between so-called “actants”, that is: specific roles (such as “subject” and “object” or “helper” and “opponent”) that are filled by people, places and events figuring in a story. Following this model, narratives can be represented as networks of interconnected concepts, which can then be subjected to structural analysis. Actantial networks have thus been inferred from large collections of textual data by means of techniques from the field of natural language processing. Tangherlini et al. have for instance extracted Greimassian networks of actants from Reddit data [16] in order to explore the structural properties of conspiracy theories, and Tuters and Willaert have visualized networks of co-occurring hashtags and named entities from Instagram data in order to explore dynamics of narrative convergence [17]. Advances in the field of human-centric artificial intelligence have likewise explicitly engaged with the question of how to connect textual data from social media and other sources with network-like representations of narratives [18]. Our study builds on these examples, and investigates how networks of associations between concepts are constructed up over sequences of Telegram messages. We are thereby specifically interested in the range of “narrative profiles” that might emerge when we consider the contribution of a channel’s messages to a thread of narrative continuity.

Previous research in narratology shows that the varieties of narrative progression in new media environments are manifold and often resist a unified or cohesive “plot” [19, p. 167]. Narratologist Marie-Laure Ryan thus theorizes that alternative forms of narrative progression might take shape in relation to the “affordances” of digital media [8, p. 515], which she defines as the “constraints and possibilities” for shaping narratives offered by a medium [20, p. 2] (for a detailed discussion of the concept, also see [21]). Ryan in particular argues that the interactivity, volatility and networking afforded by digital media might, among other things, break up the linear flow of narrative [8, p. 515]. This observation resonates with recent analyses of the narrative dimensions of social media, which have foregrounded how technologically-mediated narrative orderings support the creation of identity and subjectivity, thus also putting the perspective of users center stage. Georgakopoulou’s work in the field of “small stories” analysis for instance draws attention to the sense of narrative authenticity and immediacy associated with sequential linkages of posts, countdowns, and periodical updates [22, 23]. Page, furthermore, offers analyses of the open-ended forms of narrative linearity afforded by “episodic” social media formats such as serialized status updates, comments, or iterative revisions and corrections of webpages such as wikis [24, p. 337] (also see [25]). Yet while these studies cover a diverse range of features and platforms, they are connected in their shared reliance on a method of “close reading”. Analyses thus typically drill down to the level of individual social media posts or messages, which are selected and discussed with the aim of illustrating specific narrative sequencing principles and their effects. While this qualitative approach yields substantial case studies [24, p. 329], our investigation of Telegram’s narrative affordaces also turns to the large scale analysis of texts to engage directly with the “big data” aspect that is characteristic of social media. As such, we propose a perspective that might be referred to as a “mid-level reading” of Telegram posts [26, p. 741]: a reading that takes a “distant” perspective on narratives as networks of associations between “actants”, but that also pays closer attention to the role of platform features in the construction of these networks. The empirical object of this reading will be public Telegram channels.

### Empirical foundations: Storytelling on Telegram

Users of Telegram can approach the platform in two key ways. First, Telegram is a messaging app similar to Meta’s WhatsApp that allows users to send messages and make calls. Large communities can thereby communicate with each other by joining or creating so-called “groups”, which allow for many-to-many communication among their members. These groups can either be private, meaning only invited members can join the discussion, or public, which means they are open for everyone. Secondly, Telegram also offers one-to-many messaging “channels”, in which the owner of the channel can post messages that are visible to an audience of subscribers. As users subscribing to such channels cannot interact with their contents, nor with each other, Telegram channels can be characterized as “broadcasting tools” [27]. By analogy with Telegram groups, Telegram channels can either be made private or public.

This study specifically focuses on the narrative affordances of public Telegram channels that can be previewed from a browser. This choice is doubly motivated. First, public channels form an accessible and public-facing part of the platform, contributing to what Telegram CEO Pavel Durov refers to as the platform’s main “social networking” function [28]. As the contents of previewable public channels can be accessed by people who do not have a Telegram account, such channels might reach large audiences beyond Telegram, and as such they may have a significant impact on public discourse.

Second, public Telegram channels are marked by a relatively limited number of features. Most notably, they lack the interactivity associated with Telegram groups, where users can reply to each other’s messages and co-construct complex discourse. As such, Telegram channels make for an object of study that allows us to eliminate confounding variables related to user engagement as we connect platform features with narrative affordances. Indeed, Telegram channels are essentially one-way messaging channels in which messages posted by the channel’s owner are displayed one below the other. These messages can either be “original” texts written by the channel owner, or messages first posted elsewhere that were subsequently “forwarded” into the channel. Therefore, channels can be characterized on the basis of two key technological features: a chronological listing of messages (in which messages appear in the order they were posted), and the possibility of forwarding a message from one channel into another. Because of their similarity to profile timelines on Twitter, commentators have pointed out how this effectively makes channels a “Twitter clone” inside of Telegram, albeit without the possibility for users to reply to messages [29].

Yet while Telegram channels lack the reply structures of Twitter or the social interaction of Telegram groups (both of which can be anticipated to translate into rich forms of collaborative storytelling), some complexities still have to be taken into consideration. Building on conceptual work on platform affordances, it can first be argued that a chronological ordering of messages affords a degree of “ephemerality” [30, p. 374]. When opening a Telegram channel (e.g. in a browser), the user is presented with the most recent messages posted or forwarded by the owner. Newer messages thereby continuously push older messages upwards and out of the screen, unless the user actively scrolls up to reveal older messages. This has, among others, been referred to as the “chat-logic” of social media [30, p. 374]. Following this logic, a message’s contribution to the narrative progression in a Telegram channel occurs within a limited window of visible messages. Likewise, Telegram’s feature of message forwarding affords a degree of “shareability” of narrative content between channels [30, p. 376], whereby it can be assumed that a forwarded message somehow “fits” the narrative taking shape in the target channel.

This study is interested in linking these theoretical affordances with the *actual*, textual storytelling practices of users and communities on the platform. These uses might be broadly be referred to as the platform’s “vernaculars” [31]. In this regard, previous research has already demonstrated that Telegram is highly productive, as the platform harbours distinct antagonistic communities and narratives, including coronavirus conspiracy narratives, traces of the “Great Reset” and QAnon conspiracy theories, racist and anti-“woke” narratives, and anti-establishment and anti-government narratives [5]. The present study extends this line of inquiry by investigating whether vernacular communities with different thematic orientations on Telegram also employ distinct narrative profiles.

### Building blocks of the investigation

From previous conceptual and empirical work, it follows that our investigation of how Telegram’s features relate to profiles of narrative progression should operationalize four key elements. First, the inquiry should approach the question of Telegram’s narrative profiles from the perspective of a human reader who considers the chronological sequence of messages in a channel and attempts to infer a coherent narrative from the messages’ contents. Realistically speaking, the reader assesses the contribution of each message to this narrative in relation to a limited number of messages that precede and follow it, for instance those that might simultaneously be shown in a browser window. Second, the investigation should be based on a representation of Telegram channels that distinguishes two types of messages: messages that were originally posted in the channel, and messages forwarded into the channel from other channels. This distinction should be made in order to assess how forwarded messages fit the narrative that is being constructed in the target channel. A third element concerns the actual narrative content of each message, which, following the literature, we can conceive of as networks of “actants”. The smallest unit of analysis is thus a relation between two “actants” or concepts, which can be represented as a triple of the form (concept_1_, relation, concept_2_), e.g. (“The man”, “likes”, “cats”). The more recurring concepts a sequence of messages shares, the more narratively coherent it can be perceived to be. A final element that is to be included in the analysis is Telegram’s vernacular dimension, that is: the distinct narrative usages of the platform by different communities. These vernaculars can be revealed by adopting a comparative perspective that contrasts the narrative profiles of channels pertaining to distinct communities. On Telegram, we can define such a community as a network of channels that are interlinked on the basis of messages forwarded between them. In the ‘Data’ and ‘Methodology’ sections, we explain how we operationalize these four main building blocks of our analysis.

### Research question and hypotheses

The main objective of this study is to render visible the narrative affordances of Telegram by taking a quantitative approach to large collections of Telegram data. More specifically, we aim to uncover the narrative profiles that are afforded by Telegram’s public messaging channels. We define the narrative profile of such a channel as a frequency distribution of message types, whereby each type captures the contribution of messages to a thread of narrative continuity. By means of a a clustering analysis, we attempt to identify key patterns and trends among these narrative profiles. To this end, we draw on the aforementioned empirical observations about Telegram’s narrative contents, the concept of platform “vernaculars”, as well our conceptualization of Telegram’s narrative affordances based on [30] to formulate the following two hypotheses:

1. Telegram channels afford diverse narrative profiles, corresponding with distinct vernacular uses of the medium’s features.
2. Networks of Telegram channels sampled from thematically different seed channels lean towards distinct narrative profiles.

Our first hypothesis thus captures the more general notion that the same features of Telegram allow for diverse ways of building up a narrative. Some channel owners might for instance choose to conceive of their channel as a “content aggregator” and primarily forward messages from other channels: a practice that might result in rather disparate narrative profiles. Others might first and foremostly use their channels to post their own, original messages, which could theoretically yield a more coherent, meticulously curated narrative profile. Our second hypothesis is more granular, and takes into account theoretical and empirical work that associates certain narratives on social media with specific narrative profiles. This notably concerns the idiosyncratic narrative profile of conspiracy theories, which, as we have indicated, have been shown to circulate in Telegram channels. In the literature, the narrative dynamics of such conspiracy theories have been characterized as “singularity” in which seemingly heterogeneous concepts converge [17]. On Twitter, scientific concepts such as “mRNA” (messenger ribonucleic acid) have for instance become enveloped in narratives voicing concerns over compulsory vaccination, but also calls for a “Nuremberg 2” [32]. Building on such observations, we might expect Telegram channels pertaining to conspiracy theories to be characterized by more disparate narrative profiles, in which there is little narrative continuity between consecutive messages. Such cases might then be contrasted with channels whose profiles pose less of a challenge to traditional forms of narrative coherence and progression.

In the following sections, we test these hypotheses by analysing a dataset of 492 public Telegram channels, spanning partly overlapping datasets pertaining to cryptocurrencies, the “Great Reset” conspiracy theory, the pro-Trump conspiracy theory that claims the 2020 US presidential elections had been “stolen”, far-right countercultural content, and pro-Russian disinformation about the war in Ukraine. To this end, our methodology simulates the perspective of a reader scrolling through these channels, and deploys techniques from natural language processing to categorize each message according to its narrative “fit” within a limited window of messages. These categorizations provide the basis for an agglomerative hierarchical clustering analysis based on behavioural profiles, in which channels with similar distributions of messages over categories are clustered together, thereby revealing the main narrative profiles that characterize the data.

## Data

We analyse a dataset of 492 public Telegram channels that can be previewed from a browser (total number of messages = 2,724,187, median number of messages per channel = 1724, mean number of messages per channel = 5537). It should be acknowledged upfront that in doing so, we examine a publicly available part of Telegram that can even be accessed by viewers without an account. As such, our data have been collected in the public interest from publicly available sources, and no personal data were collected or retained in this process. However, following [5], we argue that this public availability online does not suffice as a criterion to include data such as channel names or message contents unaltered in this study. No channel names or literal message contents will therefore be shared in or along with this paper (see ‘Data Availability’ statement).

**Fig 1:**
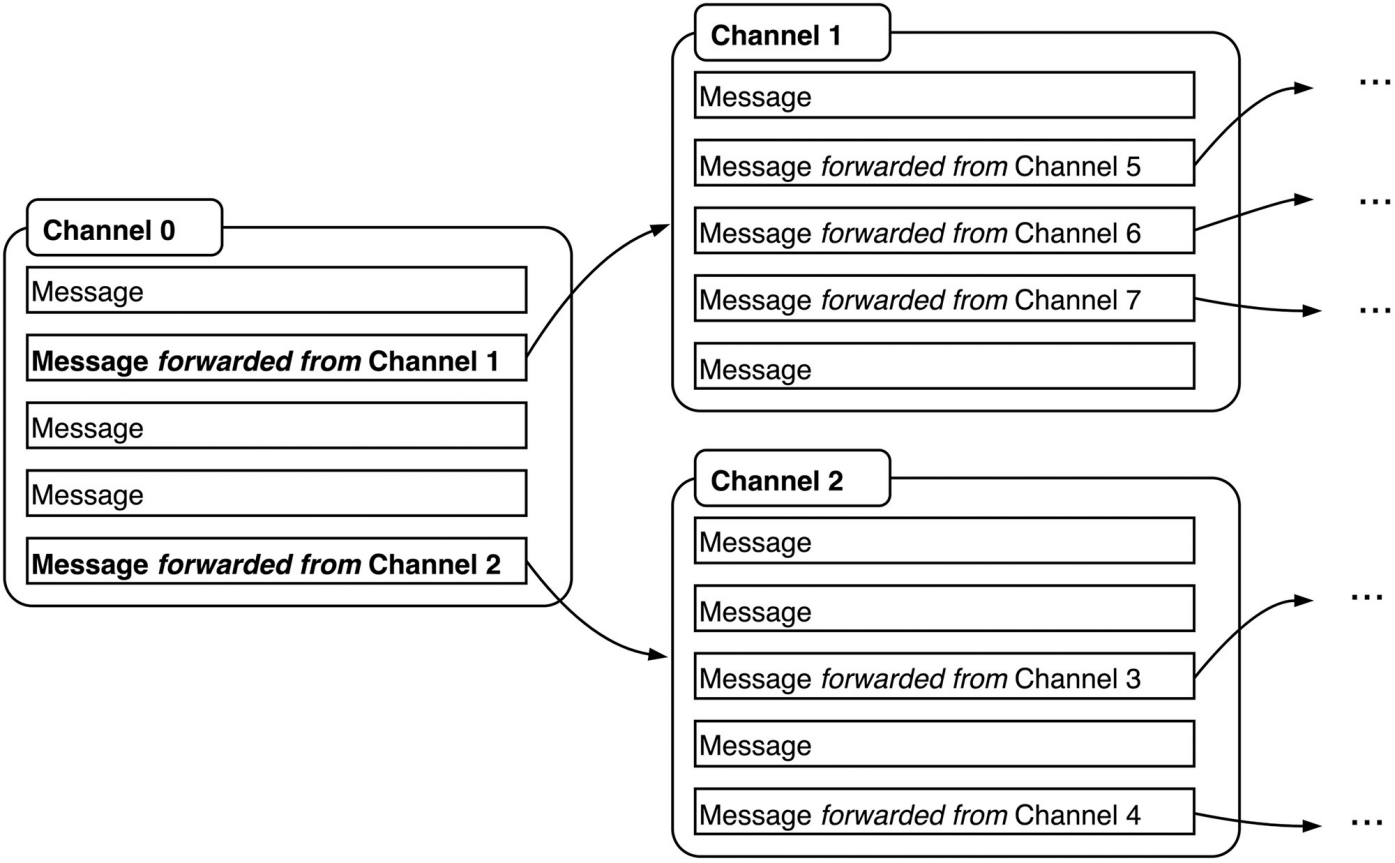
“Snowballing” method for Telegram data collection.

We collected our dataset by means of the “snowballing” method for Telegram research described in [4, 5]. As illustrated in Fig 1, this “digital methods” approach [33] repurposes Telegram’s feature of message forwarding to inductively map networks of interconnected channels. It is thus assumed that if a channel forwards a message from another channel, some meaningful connection exists between both, warranting their inclusion in the same dataset. We automate and scale up this data collection procedure by means of a purposely-built web scraper (see ‘Data Availability’ statement), which iterates over the contents of a seed channel’s public browser preview, stores its text messages, and then repeats this process for any associated channels identified on the basis of forwarded messages. For each message, we keep track of whether it constitutes an “original” message or a message forwarded from another channel. Data were thus collected from five seed channels. These were selected for their topical diversity, as well as their overall relation to disinformation and other antagonistic content that has come to be associated with Telegram discourse. Potential seed channels were first identified through Google searches for relevant keywords and through indexing websites for Telegram (e.g. [34]), and an eventual selection of five was made on the basis of qualitative assessments of the channels’ contents (see S1 File). From each of these seeds, we then “snowballed” associated channels up to a given network depth from the seed. In this process, we ran scrapers in parallel over the same time period. After this period, we preserved the data at the last fully achieved scraper depth. Due to high numbers of forwarded links in some channels and the combinatorial growth of the “snowball” this entails, the achieved scraper depth varies per dataset. For each dataset, only channels with at least 95% of message texts in English and over 200 posts after parsing were thereby retained for analysis (see ‘Data cleaning and preprocessing’ section).

Our first dataset (N = 133 channels, network depth = 4, total number of messages = 1,057,193, median number of messages per channel = 1084, mean number of messages per channel = 7949) was collected from a seed channel related to cryptocurrencies, a form of decentralized digital currencies. The channels in this dataset are concerned with different digital currencies or “coins”, such as Bitcoin, and their dynamics, including fluctuations in their values and the transfer of volumes of coins (along the blockchain).

A second dataset (N = 135 channels, network depth = 1, total number of messages = 861,556, median number of messages per channel = 2524, mean number of messages per channel = 6382) was “snowballed” from a seed channel pertaining to the “Great Reset” conspiracy theory. This conspiracy theory frames the Covid-19 pandemic as part of the world economic elite’s plan to impose global governance. Channels in this dataset are marked by, among other things, a preoccupation with vaccines and the Covid-19 pandemic.

The third dataset (N = 170 channels, network depth = 1, total number of messages = 894,239, median number of messages per channel = 2212, mean number of messages per channel = 5260) was collected from a seed channel that supports Donald Trump’s claims of a “stolen” 2020 election and that insists on reinstating the former president.

Our fourth dataset (N = 66 channels, network depth = 2, total number of messages = 113,771, median number of messages per channel = 888, mean number of messages per channel = 1724) is concerned with what can be referred to as far-right counterculture. This dataset was “snowballed” from a seed channel that collects cultural references and book readings from materials that have allegedly been banned from the mainstream.

The fifth dataset (N = 44 channels, network depth = 1, total number of messages = 284,137, median number of messages per channel = 3322, mean number of messages per channel = 6458), was collected from a seed channel that propagates a pro-Russian perspective on the war in Ukraine. These channels are, among other things, concerned with the geopolitical and military aspects of the conflict.

**Fig 2:**
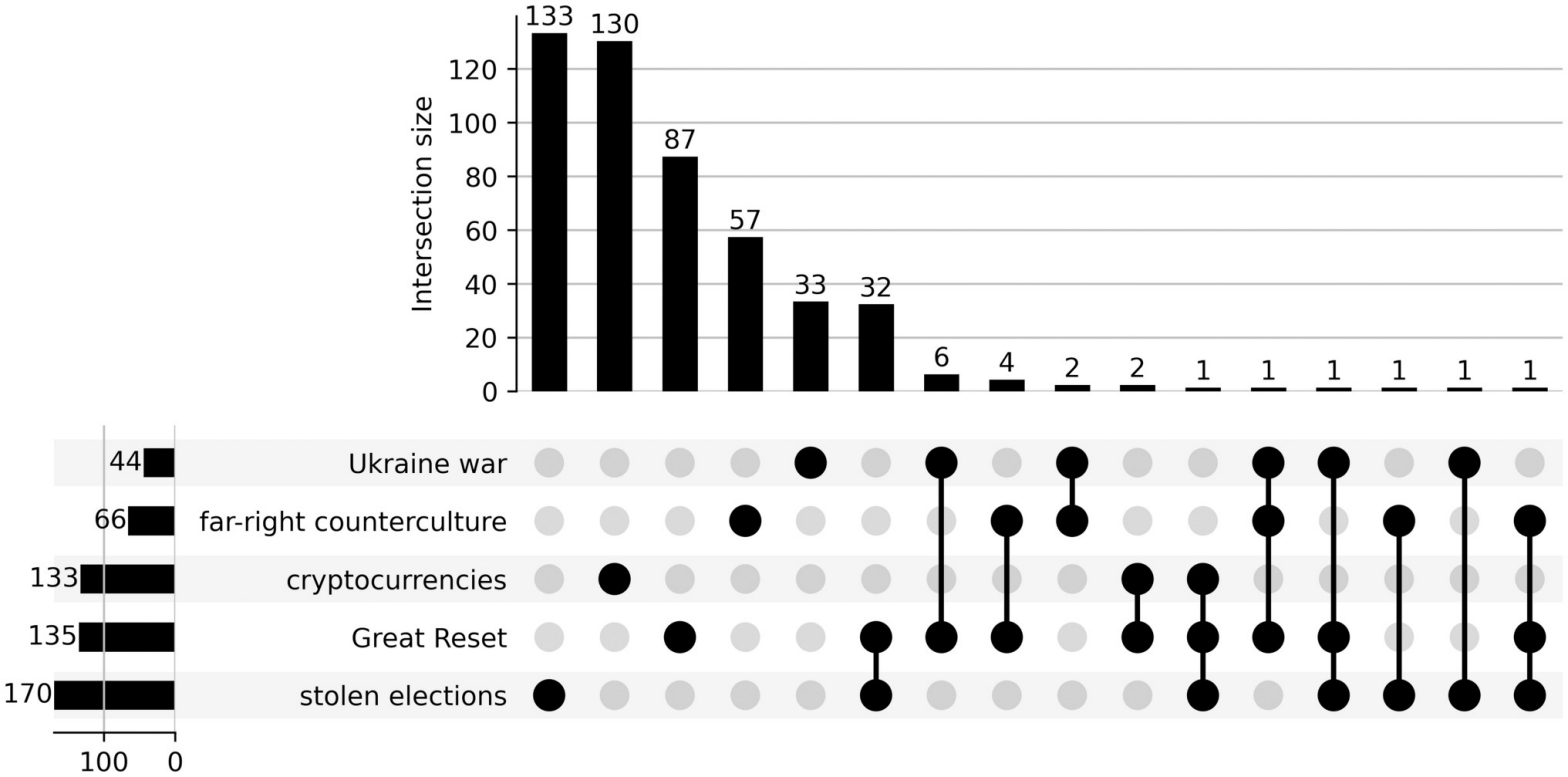
UpSetplot [35] of thematic datasets. Horizontal bars show the total number of channels in the dataset, vertical bars and connectors illustrate the overlap and size of the overlap between datasets.

As shown in Fig 2, some channels in our dataset are shared between thematic subsets. This is mainly the case for the subsets pertaining to the “Great Reset” conspiracy theory and the dataset pertaining to the “stolen election” narrative, which share 32 channels.

## Methodology

**Fig 3:**
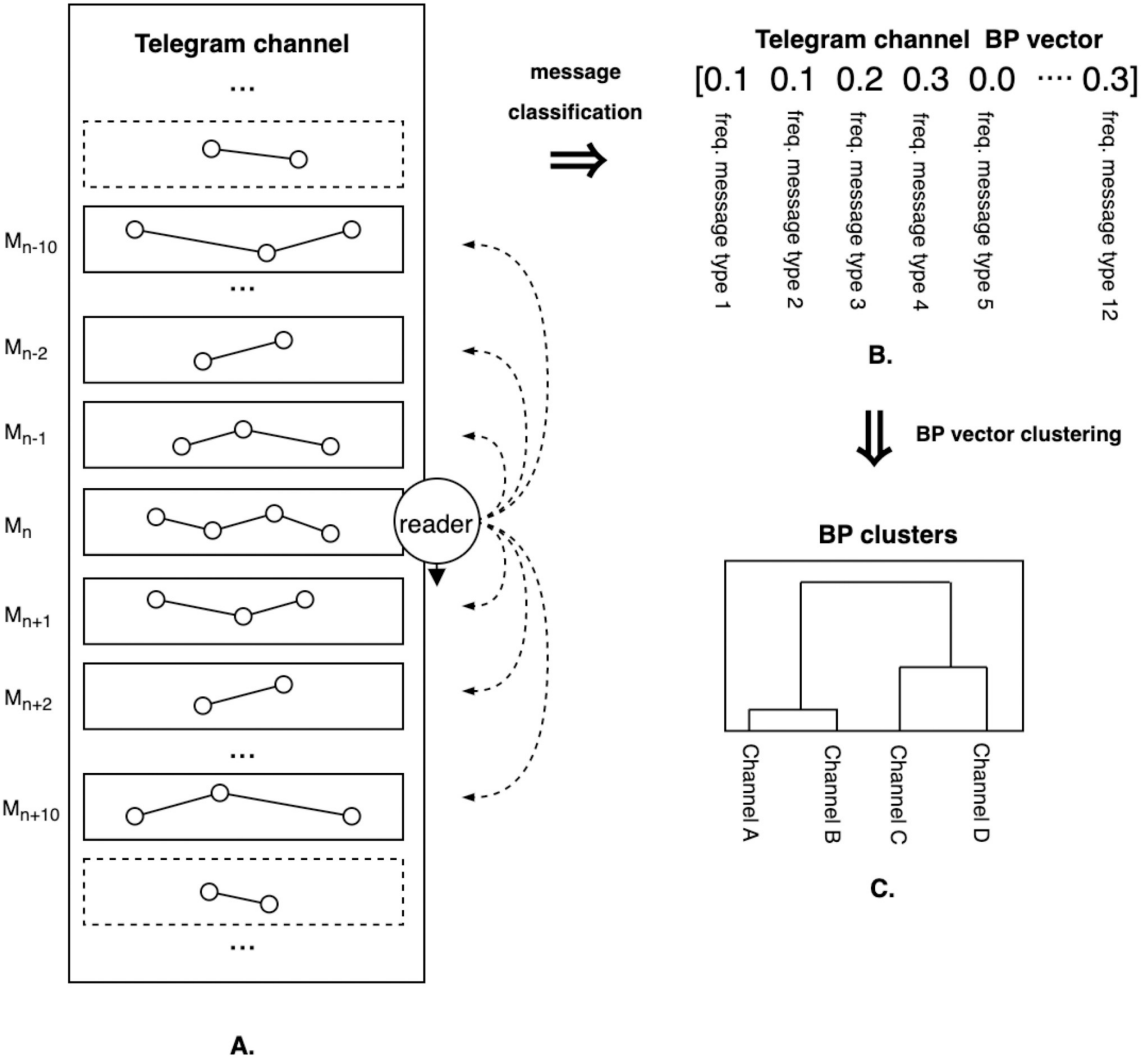
Overview of method.

We analyse the data according to a methodology that operationalizes the key elements discussed in the ‘Building blocks of the investigation’ section. Fig 3a thus shows the main components of our analysis: 1) the “reader”, who scrolls down the channel and perceives each message in order, 2) the Telegram channel, represented as a chronological sequence of original and forwarded text messages, and 3) the unfolding narrative as a series of interlinked concepts or “actants”, here represented as nodes in a network. The “vernacular” aspect of the analysis is implicit in this figure, as this aspect emerges from a comparative analysis of the five thematic datasets following the same methodology.

As the central component of the analysis, we operationalize a “reader” in the form of an NLP script that evaluates the contribution of each message to the narrative that is constructed within the window of ten messages that precede and follow it. Inspired loosely by the method presented in [36], each message is thus classified according to a predefined set of categories. A forwarded message might for instance be labelled “isolated” if it contains no overlapping concepts with the ten preceding messages, nor with the ten messages that follow it. On the other hand, a message might be labelled “emerging” if it does not share any concepts with the preceding messages, but does so with the messages that follow it. Once every message in the channel has been classified, we count the relative frequencies of message categories per type and use these values to represent the channel as a vector of features (see Fig 3b), which we will refer to as a Behavioural Profile (BP) vector. Having vectorized every channel in the dataset, we cluster the resulting behavioural profile vectors using a hierarchical agglomerative cluster analysis [37] in order to identify the diverse narrative profiles (see Fig 3c). In the following paragraphs, we discuss the details of this pipeline.

### Data cleaning and preprocessing

For each Telegram channel in the dataset, we first clean the text messages by removing emojis, hyperlinks and @-mentions. We then use the fastText language identification model [38, 39] to determine the language of each message. Only channels where at least 95% of messages are in English are retained. Next, we extract relationships between “actants” from the cleaned message texts. Following the literature, our analysis assumes that the key building blocks of narratives are syntactic relationships between concepts [40]. Each of the cleaned messages is therefore parsed using a dedicated natural language processing pipeline built with the SpaCy NLP library [41] in order to extract from the text triples of the form (concept_1_, relation, concept_2_) (see ‘Data Availability’ statement). To this end, we first split the message text into sentences (sentencization). In each sentence, we identifiy the verbs. For each retrieved verb, the dependency tree is navigated to identify the subject noun, as well as its objects, which might be labelled as direct object (“dobj”), attribute (“attr”), dative (“dative”), prepositional object (“pobj”), or agent (“agent”). As such, a sentence can contain multiple triples, e.g. the sentence “The man likes cats and dogs” will yield the triples (“The man”, “likes”, “cats”) and (“The man”, “likes”, “dogs”). These triples are then harmonized by lemmatizing nouns and verbs, and by making texts lowercase. Each Telegram message is thus represented as a series of triples. Triples containing personal pronouns (e.g. “we”, “they”) are omitted from the analysis. This is done in order to avoid skewed results due to problems with coreference [40, p. 42-43]. The pronoun “they” might for instance be repeated across multiple messages, but in each message it might refer to a different “actant”. As such, including these pronouns in the analysis is likely to yield false positives in terms of the identified narrative continuity. Messages without any triples or without remaining triples after filtering out those with pronouns are likewise removed. In the end, only Telegram channels with at least 200 remaining messages are retained for further analysis, amounting to a total of 492 unique channels. In S1 File, we provide an overview and discussion of the most frequent “actants” that figure in the data.

### Message classification

At the center of our analysis is the idea that each message in a Telegram channel can be categorized according to its contribution, or lack of contribution, to the thread of narrative continuity. Assuming that a narrative should comprise a repetition of concepts (either in the “subject” or “object” position of an extracted triple) within the scope of a given number of messages (in this case 10), messages are categorized by their contribution to a narrative as follows. For each message in the channel, we make an aggregated list of all concepts that figure in the extracted triples. Next, we make a count vector representation of each message’s list of concepts using python’s scikit-learn CountVectorizer function [42]. We then iterate over the message’s vector representations in chronological order, calculating the average cosine distance with the ten messages that precede it, and the average cosine distance with the ten messages that follow it. Finally, inspired by the analysis and concepts put forth in [36], we use each message’s average cosine distances to the ten messages preceding it and the ten messages following it as the basis for a classification of each message. We thereby consider the average distance to the messages preceding it as an expression of the message’s “novelty”, that is, how “new” the message’s core narrative concepts are in relation to those in the window preceding it. Correspondingly we consider the average distance to the messages following it as an expression of the message’s “transience”, that is, the degree to which the message’s narrative concepts disappear or fade in the messages following it. The message’s “resonance” then, is the difference between its “novelty” and its “transience”. These scores for “novelty”, “resonance” and “transience” are then used to classify each message’s contribution to the construction of narrative meaning. As we explain in more detail in S1 File, each message in our dataset is thus labelled according to one of six possible categories: “isolated”, “continued”, “emerging”, “fading”, “continued-emerging”, or “continued-fading”.

### Narrative profiles analysis

We identify the narrative profiles in the data following a method that is inspired by the behavioural profiles analysis described in [37, ch.15] and [43]. Each channel is thus represented as a vector (the BP vector) of the proportions of messages for each of the categories described in the previous section. As our analysis also aims to take into account the specific contribution of forwarded messages, we then extend this vector with the proportions for the same categories, but this time exclusively for the forwarded messages in the channel. This combined vector composed of 12 values thus allows us to capture the overall proportions of the channel’s message types, as well as those of its forwarded messages. The latter is important for assessing the relative “fit” of forwarded messages with the target channel. We then perform an agglomerative hierarchical clustering of the vectors for each dataset based on the cosine distance. Based on the approaches described in [37, p. 310-311], the Ward algorithm was selected as the main linkage method. As this algorithm minimizes the increase in variance in distance between cluster members as clusters grow, it tends to produce “compact” clusters [37, p. 311]. This granularity benefits our analysis, which is aimed at mapping the diversity of narrative profiles. A validation of this method is offered in S1 File. The optimal number of clusters was determined by calculating the silhouette widths for each number of clusters and taking the cluster number corresponding with the highest silhouette width [37, p. 309-312]. The resulting clusters were interpreted by means of plots of the average values of the vectors in each cluster.

## Findings

### Overview

Our study set out to test the double hypothesis that 1) Telegram channels afford diverse narrative profiles, corresponding with distinct vernacular uses of the platform’s features, and that 2) networks of Telegram channels sampled from thematically distinct seed channels lean towards distinct narrative profiles. The results of our analyses confirm both hypotheses.

Firstly, on the aggregate level, a behavioural profiles analysis of all 492 channels in the data reveals three distinct narrative profiles. This diversity of narrative profiles speaks to our first hypothesis. The majority of Telegram channels (70.9%) furthermore adhere to the more “disparate” of these profiles, thus challenging established notions of narrative progression. If we compare the distributions of the thematic clusters from which Telegram channels originate for each of the uncovered profiles, the “disparate” profile is marked by a high proportion of channels from the “great reset” and “stolen elections” datasets. Conversely, the more “continuous” narrative profile is marked by a high proportion of channels from the “cryptocurrencies” dataset. From this, it follows that the relation between themes and narrative profiles is not arbitrary. This observation aligns with our second hypothesis.

Secondly, on the level of each thematic dataset, a similar analysis shows that also on this more granular level, each dataset is marked by a number of distinct profiles. This further confirms our first hypothesis. Furthermore, we can discern a preference for “disparate” narrative profiles in channels pertaining to conspiracy theories and far-right counterculture, a preference for more coherent profiles in channels pertaining to cryptocurrencies, and mixed types in channels pertaining to disinformation about the war in Ukraine. This observation further supports our second hypothesis.

In the following sections, we substantiate these general findings by presenting detailed descriptions of the results of our analyses at the two levels of aggregation.

### Combined analysis

**Fig 4:**
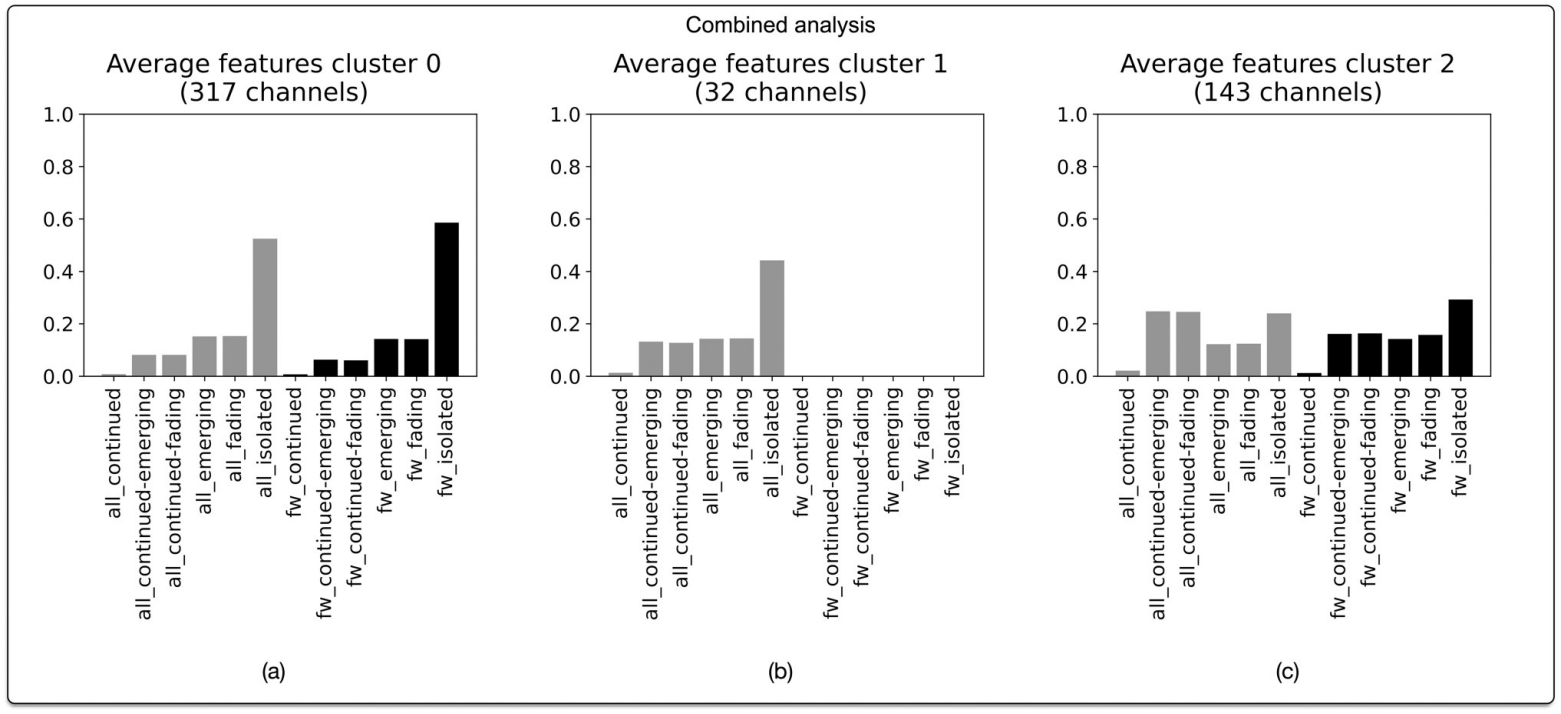
Narrative profiles for aggregated analysis of all 492 Telegram channels in the dataset. Based on agglomerative hierarchical clustering of BP vectors. Optimal number of clusters (3) corresponds with highest silhouette score of 0.49. Clustering based on cosine distance and Ward linkage method.

Our behavioural profiles analysis of all 492 channels reveals three narrative profiles. This means that for the corpus under investigation, the technical possibilities and limitations of Telegram (chronological ordering and message forwarding) afford variations on three distinct ways of constructing narratives. The defining features of each of these profiles are illustrated in Fig 4.

The first profile, which describes 317 channels in our combined dataset (64.4%), is marked by the prevalence of isolated messages, both when considering the channel as a whole and considering only forwarded messages, as well as by a relatively lower presence of continued-fading and continued-emerging messages (Fig 4a). On average, messages that are forwarded in these channels also tend to be slightly more “disparate” (and thus less fitting with the narrative that is being constructed) than expected based on the overall proportion of isolated messages. The majority of channels in the dataset, in other words, are marked by a narrative profile in which on average more than half of the messages in the channel do not bear any narrative connection to other preceding or subsequent messages within our window of analysis. This analysis is further supported by the second profile, which comprises 32 channels (6.5%) and is similar to the first one, except for a lack of forwarded messages (Fig 4c). Here as well, the majority of messages tends to be of the “disparate” kind.

The third narrative profile that can be discerned when considering our combined dataset captures 143 channels (29.1%) (Fig 4c). This profile distinguishes itself from the former two in that it is characterized by a relatively higher proportion of continued-fading and continued-emerging messages (especially when considering all message types), as well as by a relatively lower portion of isolated messages, whereby the proportion of isolated forwarded messages is slightly higher than what would be expected on the basis of the overall number of isolated messages.

From this overview, it follows that the majority of channels in our dataset (70.9%) are marked by a narrative profile that challenges established notions of narrative progression, with a smaller set of channels (29.1%) adhering more closely to such notions.

**Fig 5:**
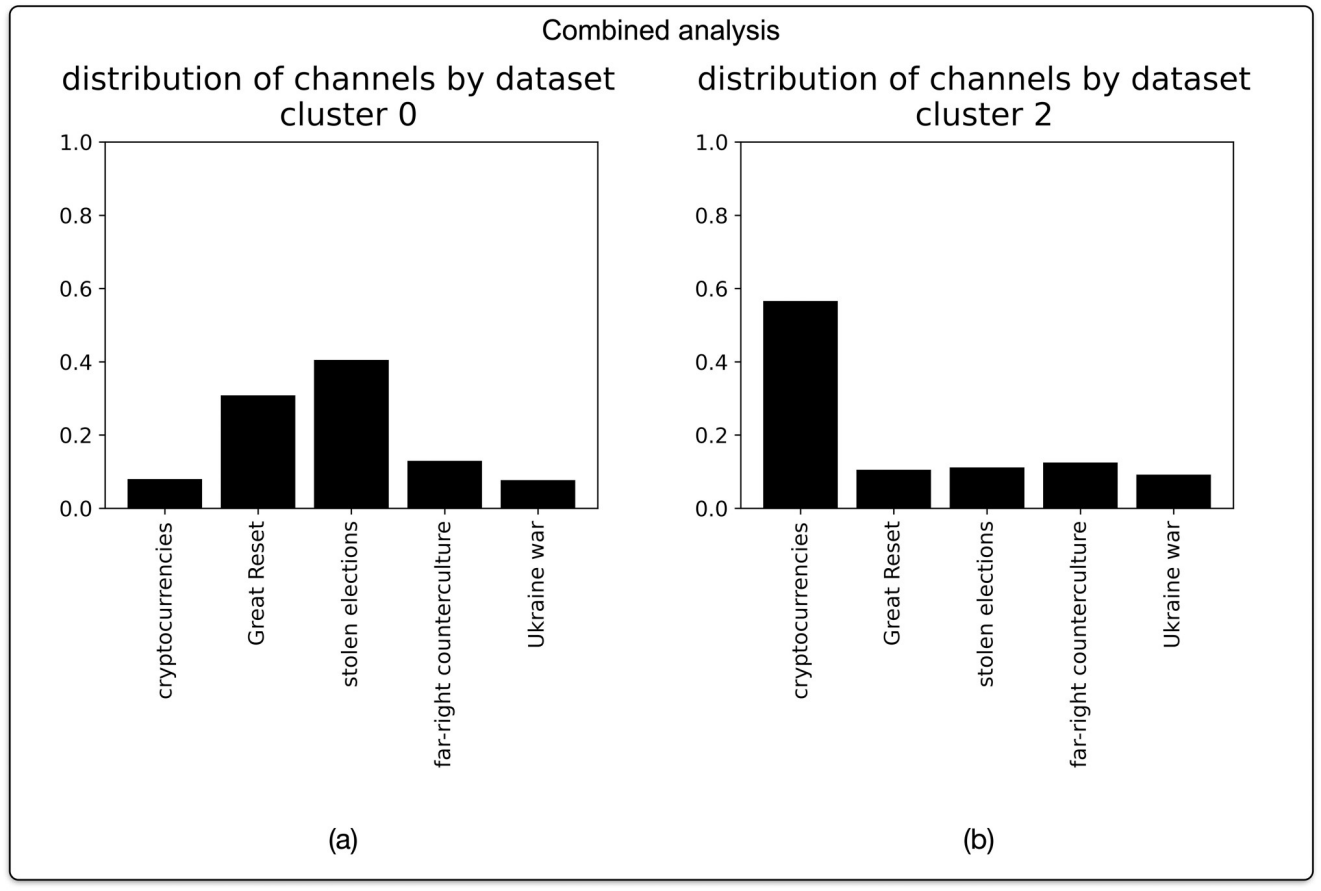
Composition of the two largest narrative profile clusters in the aggregated analysis.

As shown in Fig 5, the largest cluster of channels following the “disparate” narrative profile is composed of channels from all of our subcorpora, with slightly higher proportions of channels coming from the “great reset” and “stolen elections” datasets. The cluster of channels marked by the more “continuous” narrative profile is again composed of channels from all five subcorpora, this time with a predominance of channels from the cryptocurrencies subcorpus.

In the following section, we proceed to examine the diverse narrative profiles of each thematic subcorpus.

### Thematic analyses

#### Cryptocurrencies

**Fig 6:**
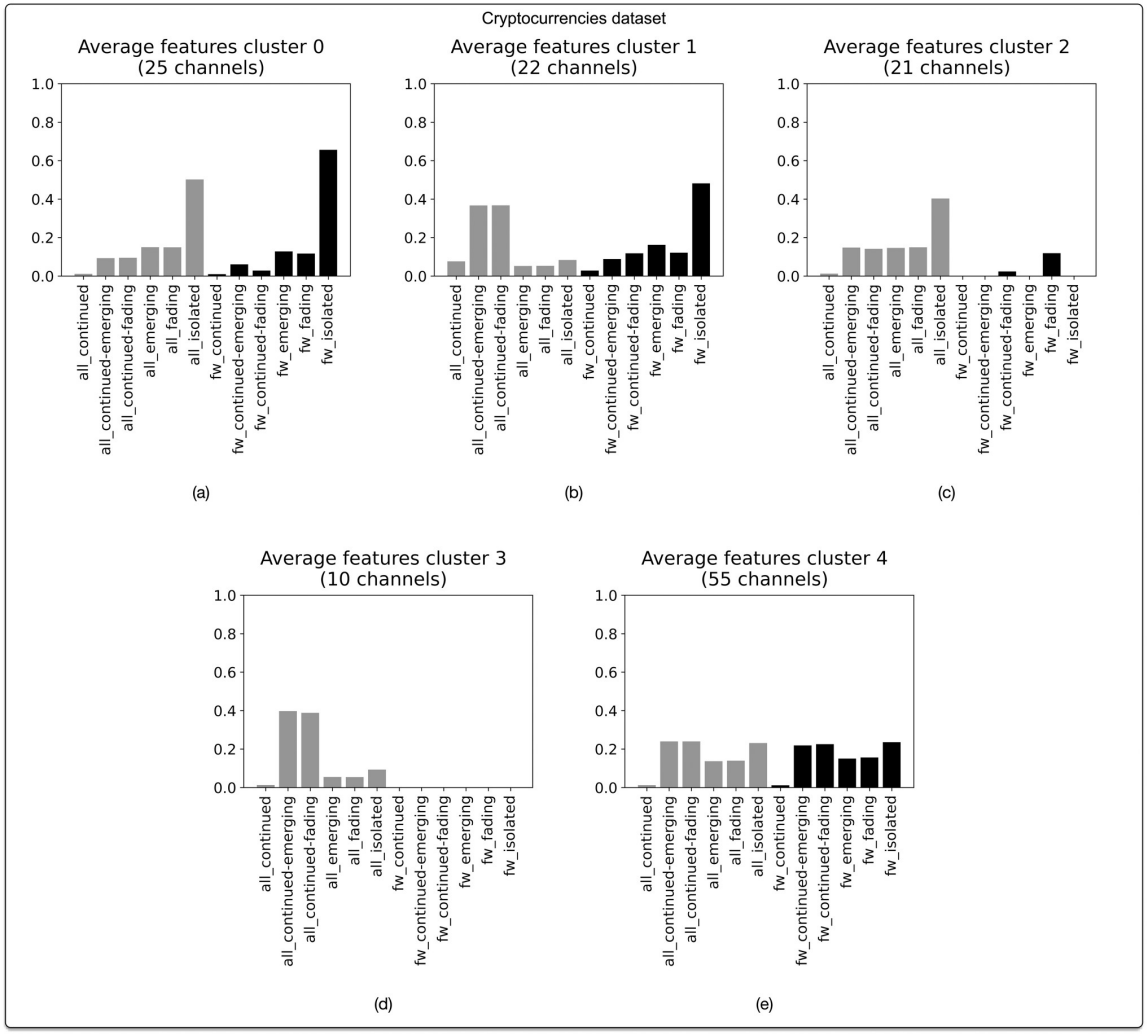
Narrative profiles for Telegram channels in the dataset pertaining to cryptocurrencies. Based on agglomerative hierarchical clustering of BP vectors. Optimal number of clusters (5) corresponds with highest silhouette score of 0.33. Clustering based on cosine distance and Ward linkage method.

As shown in Fig 6, five narrative profiles can be discerned in our subcorpus of Telegram channels pertaining to cryptocurrencies (N = 133 channels). A first profile marking 25 channels (18.8%) closely resembles the “disparate” profiles that were revealed on the aggregate level (Fig 6a). In relation to the other profiles, this profile is marked by the prominence of isolated messages (both when we consider all messages combined and when examining only forwarded messages). Forwarded messages in these channels tend to be more isolated than what would be expected based on the global number of isolated messages. Along similar lines, we can identify a profile marking 21 channels (15.8%) that is defined by a predominance of isolated messages, and a lack of forwarded messages (Fig 6b). Overall, 34.6% of channels in this dataset is thus characterized by an apparent lack of narrative continuity. Over half of the channels (65.4%) are distributed across three narrative profiles that are characterized by a higher degree of continuity, that is, an overall lack of isolated messages and a relative prominence of messages that extend the thread of narrative continuity. Illustrative here is the profile comprising 55 channels (Fig 6e), which is marked by predominantly “continued” message types. This predominance of “continued” narrative profiles is clearly marked in relation to the distribution of profiles that was observed in our combined analysis.

#### Stolen elections

**Fig 7:**
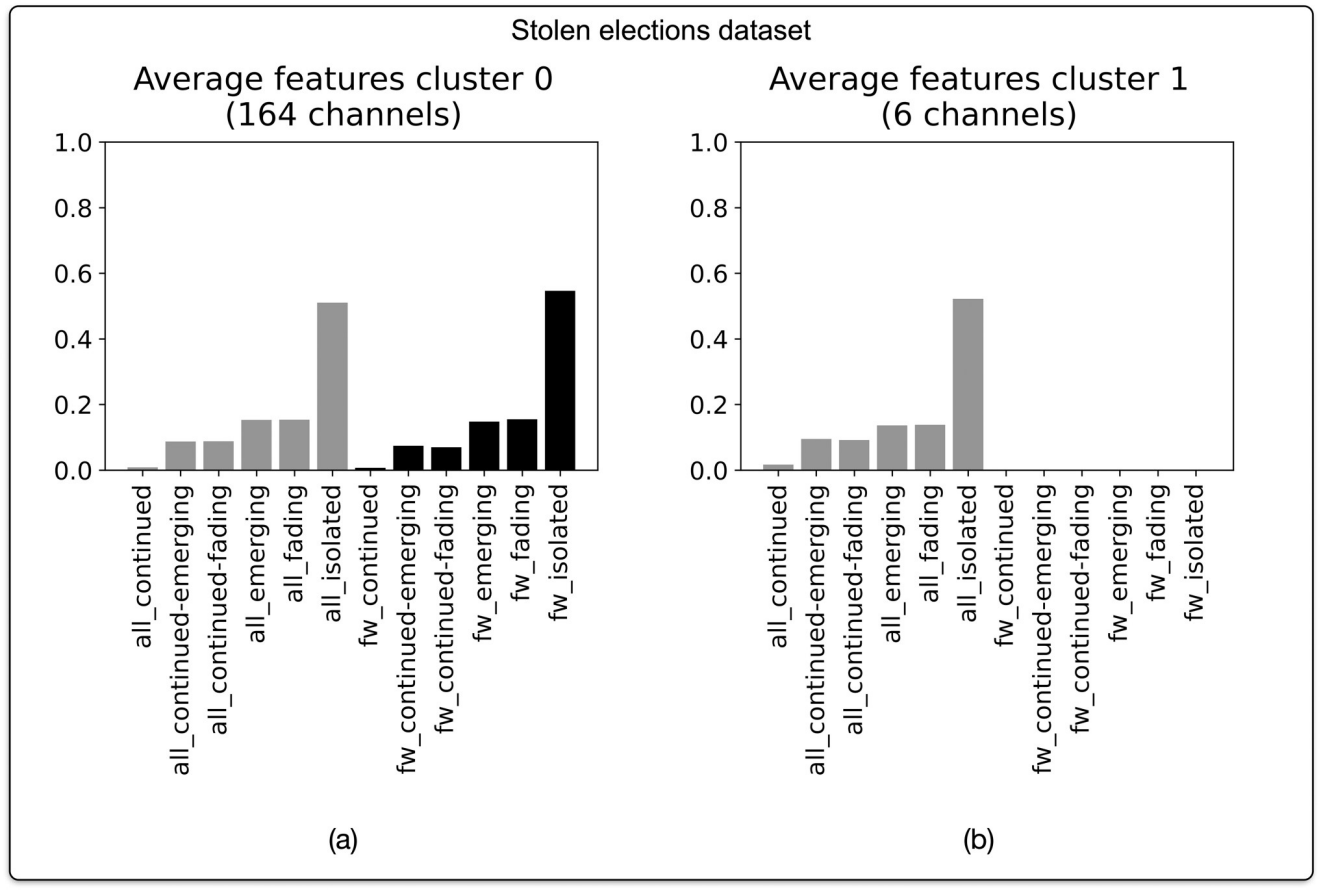
Narrative profiles for Telegram channels in the dataset pertaining to the stolen 2020 election conspiracy theory. Based on agglomerative hierarchical clustering of BP vectors. Optimal number of clusters (2) corresponds with highest silhouette score of 0.49. Clustering based on cosine distance and Ward linkage method.

This predominance of “continued” narrative profiles in the cryptocurrencies dataset can be contrasted with the prevalence of “disparate” narrative profiles in the stolen elections dataset (N = 170 channels) (Fig 7). 164 channels in this dataset follow the prototypical “isolated” profile uncovered in our dataset as a whole (Fig 7a), with the remaining 6 channels following a similar pattern lacking forwarded messages (Fig 7b). This means that in our narrative profiles analysis of this dataset, 100% of channels can be classified according to a “disparate” profile. This predominance of channels with a “disparate” narrative profile is clearly marked in relation to the distribution of narrative profiles that was observed in our combined analysis of all datasets.

#### Far-right counterculture

**Fig 8:**
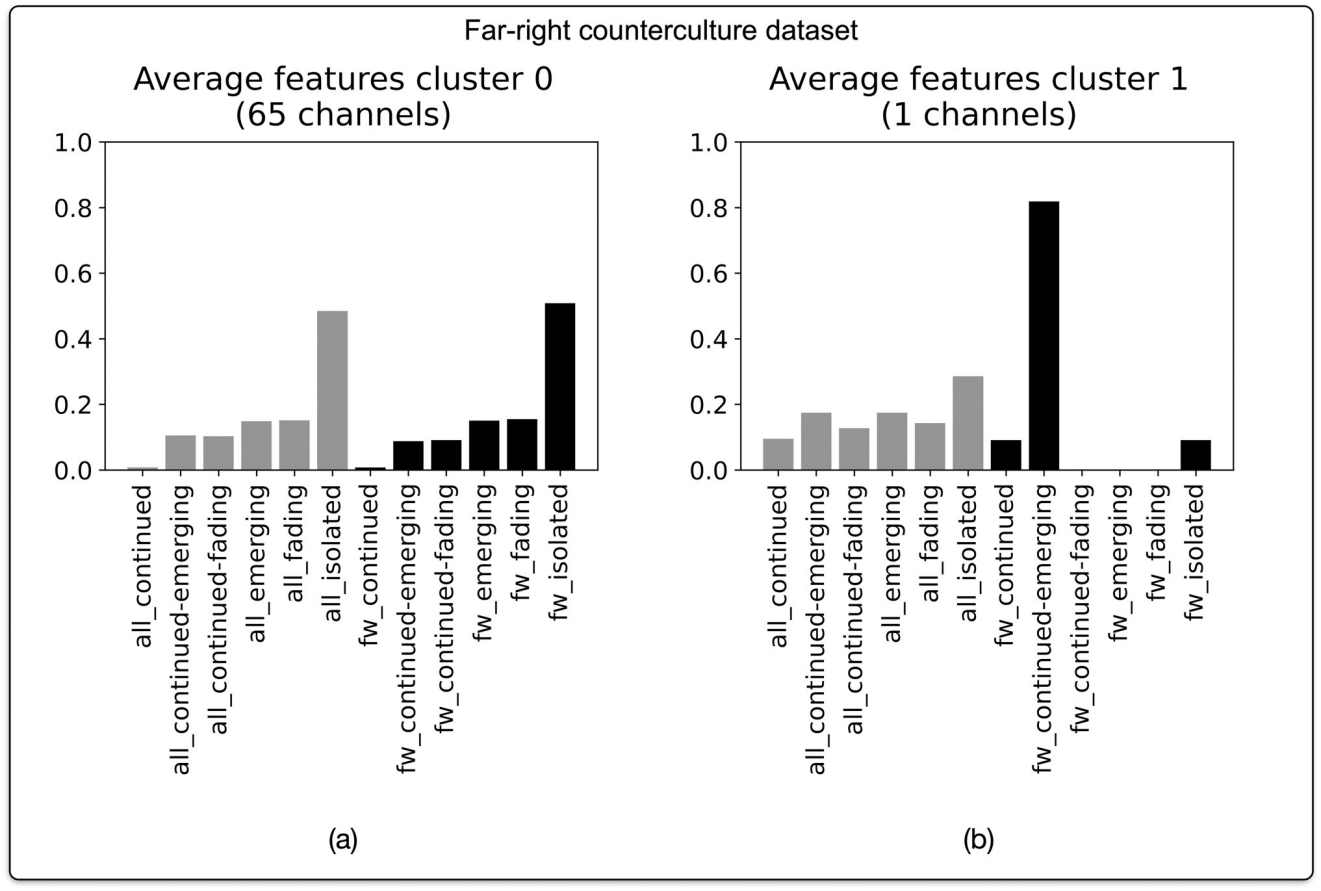
Narrative profiles for Telegram channels in the dataset pertaining to far-right counterculture. Based on agglomerative hierarchical clustering of BP vectors. Optimal number of clusters (2) corresponds with highest silhouette score of 0.42. Clustering based on cosine distance and Ward linkage method.

Similar to the aforementioned dataset about alleged election fraud, the dataset snowballed from the seed channel pertaining to counterculture (N = 66 channels) is marked by a “disparate” narrative profile (see Fig 8).

#### The “Great Reset”

**Fig 9:**
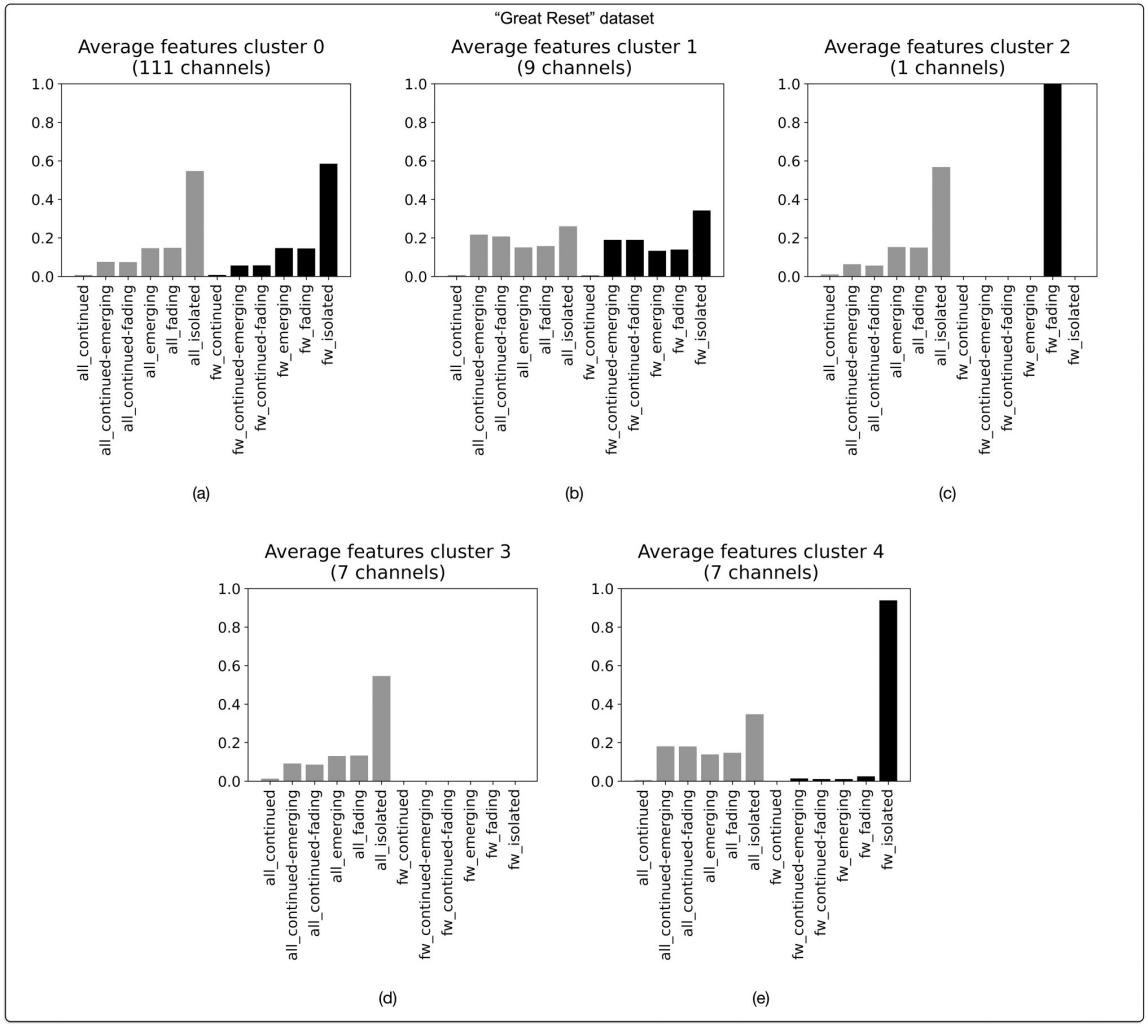
Narrative profiles for Telegram channels in the dataset pertaining to the “Great Reset” conspiracy theory. Based on agglomerative hierarchical clustering of BP vectors. Optimal number of clusters (5) corresponds with highest silhouette score of 0.43. Clustering based on cosine distance and Ward linkage method.

Likewise, the dataset of channels snowballed from a seed channel pertaining to the “Great Reset” conspiracy theory (N = 135 channels) is characterized by a large number of channels that have a narrative profile marked by isolated messages (Fig 9). 111 channels (82.2%) in this dataset have a profile that is reminiscent of the main “disparate” profile uncovered in all datasets combined (Fig 9a). To this can be added 7 channels that have a similar profile, but that lack forwarded messages (Fig 9d), and another 7 that are marked by the high number of “isolated” forwarded messages (Fig 9e). Only 9 channels (6.7%) have a typical “continued” profile (Fig 9b).

#### Ukraine war

**Fig 10:**
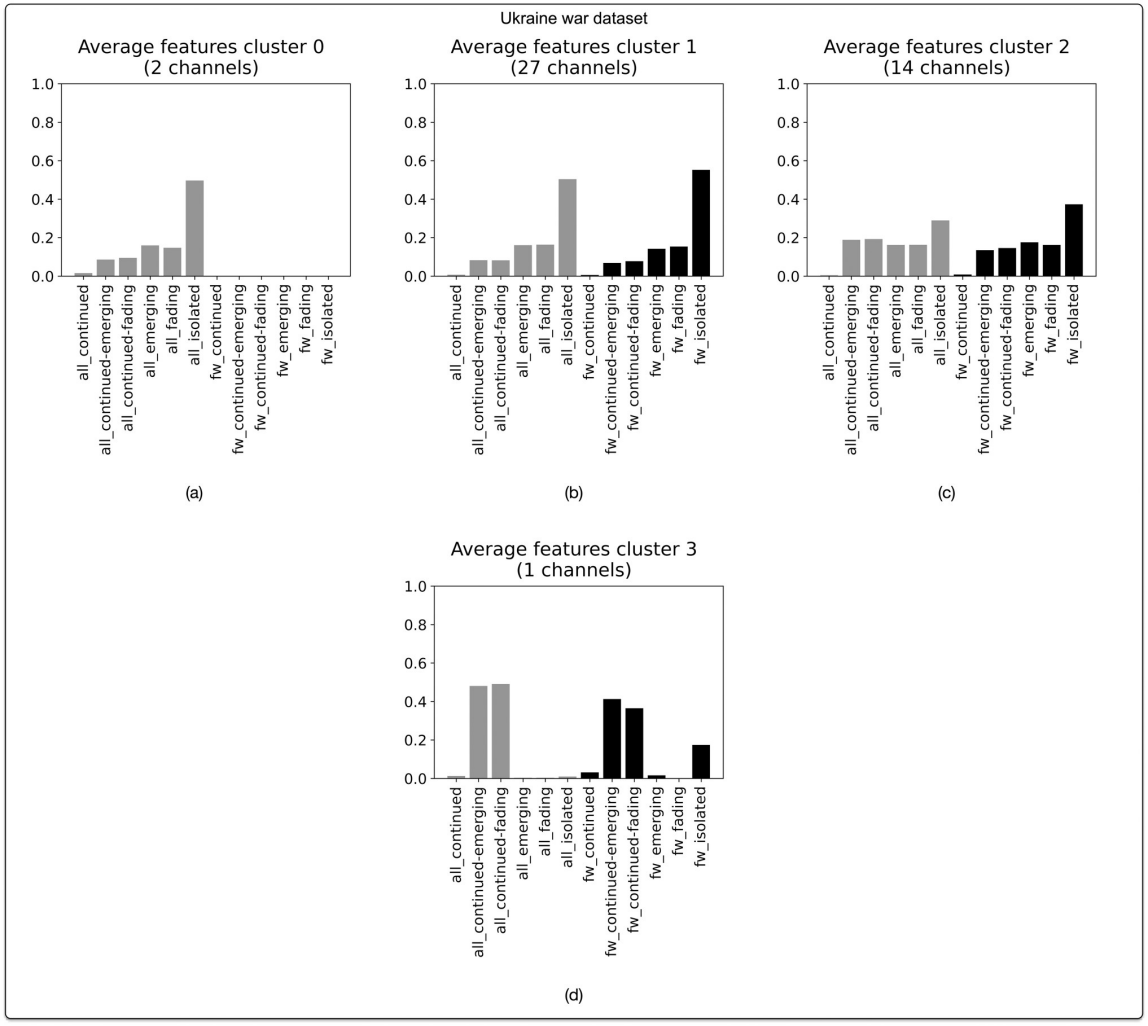
Narrative profiles for Telegram channels in the dataset pertaining to the war in Ukraine. Based on agglomerative hierarchical clustering of BP vectors. Optimal number of clusters (4) corresponds with highest silhouette score of 0.46. Clustering based on cosine distance and Ward linkage method.

Our dataset snowballed from a seed channel pertaining to disinformation about the war in Ukraine (N = 44 channels) contains 27 channels (61.4%) that follow the “prototypical” disparate pattern (Fig 10b), along with 2 channels (4.5%) that have a similar pattern but without forwarded messages (Fig 10a). 14 Channels (31.8%) are marked by the “continuous” profile (Fig 10c). With this distribution of profiles, the dataset can be situated in between the “cryptocurrencies” dataset and the datasets relating to disinformation and counterculture.

## Discussion

Previous literature on platform affordances and empirical research on Telegram allows us to connect the features of public Telegram channels with three affordances at the intersections of social interaction and text construction. First, on a technical level, Telegram channels consist of chronological listings of messages. As we have established, this affords a degree of “ephemerality”, where messages are likely to be considered within the limited window of what can be shown on screen. Secondly, Telegram’s functionality of forwarding messages from one channel into another allows for a form of social networking, where channels forwarding each other’s messages become connected on the grounds of shared interests. This likewise enables a limited form of collaborative posting in which a channel’s owner can introduce content from other channels into their own sequence of original messages. Third, on the level of platform governance, Telegram’s promise of lenient content moderation has been shown to sustain antagonistic vernacular communities and idiosyncratic narratives on the platform. Combined, these elements allow us to describe how sequences of messages on Telegram are constructed and perceived, as well as how a wide range of communities and contents are tolerated on the platform. Our present analysis builds on this state of the art and moves beyond it by putting the question of narrative center stage, and by providing empirically-informed insights into the possibilities for storytelling that emerge from the aforementioned platform features and affordances.

An aggregated analysis of all 492 channels in our dataset thus finds that public Telegram channels afford variations of three distinct narrative profiles. The first profile is one where messages contribute to the construction of a continuous narrative. The second, more prevalent profile is characterized by the predominance of “isolated” messages that bear little contribution to a coherent narrative. A third, less prominent profile aligns with the latter, but is marked by the absence of forwarded messages. This finding confirms our first hypothesis. It furthermore speaks to our second hypothesis that when we examine the narrative profiles of our five datasets separately, we find that, depending on the dataset, certain profiles are preferred over others. We can thus discern a preference for disparate profiles (marked by a prevalence of “isolated” messages) in channels pertaining to conspiracy theories and far-right counterculture, a preference for coherent profiles (marked by a relative absence of “isolated” messages) in channels pertaining to cryptocurrencies, and mixed types in channels pertaining to disinformation about the war in Ukraine. Our study thus empirically demonstrates that a seemingly simple set of platform features still presents users with diverse ways of telling stories.

Furthermore, it is a key finding of our study that the most frequent narrative profile in our dataset is a “disparate”’ one that challenges traditional notions of narrative coherence. This finding can in part be attributed to the operationalization of our analysis, which maintains a rather rigorous definition of what constitutes a message’s contribution to narrative continuity. Yet this does not fully explain the vernacular trends that we observe in the data. We must therefore explore the possibility that for those channels with a “disparate” narrative profile, some alternative form of narrative cohesion effectively has to be sought on other levels than those of a repetition of concepts or “actants” across sequences of messages [19].

In this regard, our finding that Telegram channels pertaining to conspiracy theories are marked by “disparate” narrative profiles align with previous observations about the idiosyncratic and dynamic nature of conspiracy narratives online. It has for instance been shown that such narratives are marked by the convergence of seemingly disparate actors and concepts. Following this line of reasoning, it might be proposed that what binds the messages in Telegram channels pertaining to conspiracy theories is an over-arching “metanarrative” with which the reader is assumed to be familiar. One such “metanarrative” is the notion that some “globalist elites” are secretly running the world, which can manifest itself in various associations between “actants” [17]. While an in-depth, qualitative discussion of channel contents is beyond the scope of this investigation, traces of such over-arching metanarratives can be discerned among the frequency lists of actants provided in S1 File. Among the most frequent actants in our data we find people and organisations pertaining to government, the law, business, and politics. This includes recurring antagonists of international conspiracy theories, such as tech billionaire and philantropist Bill Gates (who ranks amongst the top 350 actants in our “Great Reset” and “stolen elections” datasets), and businessman and philantropist George Soros (who is in the top 400 actants of the “stolen elections” dataset). Likewise, we find that the figure of “Q”, the protagonist of the pro-Trump QAnon conspiracy theory, ranks amongst the 300 most frequent actants in the “Great Reset” dataset, and amongst the top 75 most frequent actants in the “stolen elections” dataset. More abstract actants such as “elites” are ranked among the top 600 most frequent entities across our datasets, except for the dataset on cryptocurrencies. “Globalists” are likewise ranked in the top 2000 actants across all datasets except the one on cryptocurrencies. Observations such as these suggest that anti-elite metanarratives of different kinds, including some established conspiracy theories, are indeed present in the data, where they may act as an interpretative framework that ties together some of the “disparate” narrative profiles we have detected.

Yet, it can be argued that even those channels that adhere to a “coherent” narrative profile as we have defined it here, might do so in a rigourous way that again renders them idiosyncratic from a narrative standpoint. Channels pertaining to cryptocurrencies for instance offer continuous updates on the status of a select number of cryptocurrency “coins”, including on the rise and fall of their value, as well as on the transfer of coins between so-called “wallets”. This results in a highly specialized and intensely serialized kind of narrative, not unlike the one that could be found when “reading” a ticker tape with stock information.

These empirical findings thus invite us to further theorize how platform features allow users to construct and shape narratives online, and to consider more intensively the role that platform features might have in shaping narratives. While the relatively simple features of public Telegram channels have allowed us to map some elementary narrative dynamics, it remains to be seen to what extent our findings can be generalized to other platforms. One additional, yet fundamental aspect to consider here, are the collaborative dynamics afforded by the many-to-many communication in Telegram groups, or the narrative dimensions added by the possibilities of interacting with messages through replies or comments on platforms including Twitter, Facebook and Instagram. On Twitter, for example, narratives might be constructed jointly by multiple users who reply to each other’s messages, who provide commentaries to previous tweets when reposting them, or who associate their message with those of others through shared hashtags. Likewise, Instagram and Facebook allow users to (publically) engage with other users’ posts through commenting features. This collaborative aspect is not explicitly present in public Telegram channels, where even though narratives can be constructed from messages forwarded from other channels, it can be assumed that in the end, a single agent (the channel owner) is responsible for the selection of these messages.

Online settings in which multiple agents are involved in the construction of narratives have been productively examined through the lens of network theory. Social media are thereby viewed as instances of “networked cultures”, in which multiple users participate in processes of narrative construction and meaning-making by continuously “sampling”, “remixing” and “remaking” the texts of others [44, p. 486]. In the strong version of this argument, the network-like structures of connections between users on social media are assumed to “fundamentally shape [the] form, dissemination and evolution” of conspiracy theories and other narratives [45, p. 497]. While such networks might be expansive and complex, they are also, to an extent, governed by recurring structuring processes. Examples include the Matthew principle, in which actors in the network that already have many connections tend to attract more interactions, and forms of “clustering”, where people connect with those most similar to them or most close to them [45, p. 499]. Following these principles, it might be hypothesized that also in social media characterized by many-to-many forms of communication, specific thematic thematic clusters marked by specific forms of storytelling might emerge. The extent to which these clusters might correspond with the associations between narrative profiles and themes identified in this paper, however, remains a question to be examined in future work.

## Conclusions and avenues for future research

The analysis reported on in this paper constitutes a twofold contribution to the study of Telegram’s narrative affordances.

First, on the level of empirics, our computational analysis reveals that Telegram’s features afford diverse narrative profiles, and that the preferred profiles within a dataset of Telegram channels corresponds with the thematic orientation of that dataset. These empirical observations contribute to the two main bodies of literature against which this study positions itself. For one thing, our “mid-level” reading produces new traces of narrative profiles, thus extending our knowledge of the narrative affordances of social media, especially as they are studied and theorized in the field of narratology. Here, the study corroborates previous observations about the narrative complexity of social media. For another, our empirical findings contribute to on-going discussions and investigations about the societal implications of social media. Here, we have shown that Telegram harbours intricate and emerging forms of storytelling that have yet to be explored in more detail, in particular with regards to conspiracy theories and other forms of disinformation.

Secondly, the computational approach used here to render visible the interactions between platform features and narratives constitutes a methodological contribution. On a general level, the paper proposes a machine-guided reading method that is attuned to both broader narrative patterns as well as the particularities of the platform’s affordances. This method moves beyond aggregated analyses of platform contents in that it mimics the perspective of a human reader who considers the narrative contributions of a message within a limited window of surrounding messages.

The empirical analysis and methods presented in this paper can be elaborated along a series of avenues for future research. First, the scope of the investigation might be expanded to include Telegram’s multimodal narratives, that is: narratives that comprise texts, images, and other media. Such an approach would reduce some of the abstraction introduced by the current operationalization, which focuses exclusively on text messages, and more specifically those text messages that actually contain syntactically-expressed relations between concepts. Secondly, technical limitations with regards to this textual analysis should be overcome, with future work moving beyond syntactical analyses. This might include the introduction of semantics, reasoning and other forms of “narrative understanding” as they are currently being developed in the field of artificial intelligence [46]. This also includes the use of language models beyond those developed for English. Finally, and crucially, future work on narratives and storytelling on social media might examine the role of interactions between users. This might involve cross-platform analyses to evaluate how the narrative profiles of public Telegram channels relate to those of Telegram groups (in which multiple users might contribute to the construction of narratives through chat-like interactions), as well as those of other interactive social media such as Twitter, Facebook and Instagram. Likewise, going beyond the boundaries of single platforms, cross-temporal analyses might be conducted to investigate the relation between narratives and wider societal dynamics. To this end, the reading method that has been proposed here might be expanded with further quali-quantitative methods that take into account the wider societal and cultural contexts of social media.

## Supporting information

S1 File This document provides additional information with regards to the paper’s 1) message classification procedure, 2) retrieved “actants”, 3) seed channels used for “snowballing” the datasets, and 4) clustering method. (PDF)

## References

[1] Lewandowsky S, Pomerantsev P. Technology and Democracy: a Paradox Wrapped in a Contradiction Inside an Irony. Memory, Mind & Media. 2022;1:1–9. 10.1017/mem.2021.7 PMC7613775 36415623

[2] Rogers R, editor. The Propagation of Misinformation in Social Media: A Cross-platform Analysis. Amsterdam: Amsterdam University Press; 2023.

[3] Rogers R. Deplatforming: Following Extreme Internet Celebrities to Telegram and Alternative Social Media. European Journal of Communication. 2020;35(3):213–229. doi: 10.1177/0267323120922066

[4] Peeters S, Willaert T. Telegram and Digital Methods. Mapping Networked Conspiracy Theories through Platform Affordances. M/C Journal. 2022;25. doi: 10.5204/mcj.2878

[5] Willaert T, Peeters S, Seijbel J, Van Raemdonck N. Disinformation Networks: A Quali-Quantitative Investigation of Antagonistic Dutch-speaking Telegram Channels. First Monday. 2022;27(5).

[6] Simon M, Welbers K, Kroon AC, Trilling D. Linked in the Dark: A Network Approach to Understanding Information Flows within the Dutch Telegramsphere. Information, Communication & Society. 2022;0(0):1–25. doi: 10.1080/1369118X.2022.2133549

[7] Lorenz-Spreen P, Oswald L, Lewandowsky S, Hertwig R. A Systematic Review of Worldwide Causal and Correlational Evidence on Digital Media and Democracy. Nature Human Behaviour. 2023;7(11):74–101. doi: 10.1038/s41562-022-01460-1 36344657 PMC9883171

[8] Ryan ML. Narrative and Digitality. Learning to Think with the Medium. In: Phelan J, Rabinowitz PJ, editors. A Companion to Narrative Theory. No. 33 in Blackwell Companions to Literature and Culture. Malden, MA, USA: Blackwell Publishing; 2005. p. 515–528.

[9] Venturini T, Bounegru L, Gray J, Rogers R. A Reality Check(list) for Digital Methods. New Media & Society. 2018;20(11):4195–4217. doi: 10.1177/1461444818769236

[10] Underwood T. Distant Horizons: Digital Evidence and Literary Change. Chicago and London: University of Chicago Press; 2019.

[11] Moretti F. Distant Reading. London and New York: Verso; 2013.

[12] Mani I. Computational Narratology. In: Hühn P, Meister JC, Pier J, Schmid W, editors. The Living Handbook of Narratology. Hamburg: Hamburg University; 2013. Available from: https://www-archiv.fdm.uni-hamburg.de/lhn/node/43.html.

[13] Finlayson MA. Inferring Propp’s Functions from Semantically Annotated Text. The Journal of American Folklore. 2016;129(511):55–77. doi: 10.5406/jamerfolk.129.511.0055

[14] Greimas A. Actants, Actors and Figures. In: Perron PJ, Collins FH, editors. On Meaning: Selected Writings in Semiotic Theory. Theory and History of Literature. Minneapolis: University of Minnesota Press; 1987. p. 106–120.

[15] Herman D. Actant. In: Herman D, Jahn M, Ryan ML, editors. Routledge Encyclopedia of Narrative Theory. London and New York: Routledge; 2005. p. 1–2.

[16] Tangherlini TR, Shahsavari S, Shahbazi B, Ebrahimzadeh E, Roychowdhury V. An Automated Pipeline for the Discovery of Conspiracy and Conspiracy Theory Narrative Frameworks: Bridgegate, Pizzagate and Storytelling on the Web. PLOS ONE. 2020;15(6):1–39. doi: 10.1371/journal.pone.0233879 32544200 PMC7297331

[17] Tuters M, Willaert T. Deep State Phobia: Narrative Convergence in Coronavirus Conspiracism on Instagram. Convergence. 2022;28(4):1214–1238. doi: 10.1177/13548565221118751

[18] Steels L, editor. Foundations for Meaning and Understanding in Human-Centric AI. Venice: Venice International University; 2022.

[19] Richardson B. Beyond the Poetics of Plot. Alternative Forms of Narrative Progression and the Multiple Trajectories of *Ulysses*. In: Phelan J, Rabinowitz PJ, editors. A Companion to Narrative Theory. No. 33 in Blackwell Companions to Literature and Culture. Malden, MA, USA: Blackwell Publishing; 2005. p. 167–180.

[20] Ryan ML, editor. Narrative across Media. The Languages of Storytelling. Frontiers of Narrative. Lincoln and London: University of Nebraska Press; 2004.

[21] Bucher T, Helmond A. The Affordances of Social Media Platforms. In: Burgess J, Marwick A, Poell T, editors. The SAGE Handbook of Social Media. London: SAGE Publications Ltd; 2018. p. 233–253.

[22] Georgakopoulou A. Small Stories as Curated Formats on Social Media: The Intersection of Affordances, Values & Practices. System. 2021;102:102620. doi: 10.1016/j.system.2021.102620

[23] Georgakopoulou A. Co-opting Small Stories on Social Media: A Narrative Analysis of the Directive of Authenticity. Poetics Today. 2022;43(2):265–286. doi: 10.1215/03335372-9642609

[24] Page R. The Narrative Dimensions of Social Media Storytelling. Options for Linearity and Tellership. In: De Fina A, Georgakopoulou A, editors. The Handbook of Narrative Analysis. Chichester: John Wiley & Sons, Inc.; 2015. p. 329–347.

[25] Page R. Re-examining Narrativity: Small Stories in Status Updates. Text & Talk. 2010;30(4):423–444. doi: 10.1515/text.2010.021

[26] Felski R. Latour and Literary Studies. PMLA/Publications of the Modern Language Association of America. 2015;130(3):737–742. doi: 10.1632/pmla.2015.130.3.737

[27] Telegram. Telegram Frequently Asked Questions;. Available from: https://telegram.org/faq#q-what-is-telegram-what-do-i-do-here.

[28] Durov P. Telegram message on 23 December 2020 by Pavel Durov; 2020. Available from: https://t.me/s/durov/142.

[29] Lobao M. Telegram v3.2 Brings Channels For Broadcasting Your Messages To The World; 2015. Available from: https://www.androidpolice.com/2015/09/22/telegram-v3-2-brings-channels-broadcasting-messages-world/.

[30] Van Raemdonck N, Pierson J. Conceptueel Kader voor Wisselwerking van Platformkenmerken, Affordances en Normen op Sociale Media. Tijdschrift voor Communicatiewetenschap. 2022;50:358–383. doi: 10.5117/TCW2022.4.005.RAEM

[31] Peeters S, Tuters M, Willaert T, de Zeeuw D. On the Vernacular Language Games of an Antagonistic Online Subculture. Frontiers in Big Data. 2021;4:718368. doi: 10.3389/fdata.2021.718368 34447929 PMC8383811

[32] Tuters M, Willaert T, Meyer T. How Science Gets Drawn Into Global Conspiracy Narratives. Issues in Science and Technology. 2023;39(12):32–36. doi: 10.58875/POZR1536

[33] Rogers R. Digital Methods. Cambridge, MA: MIT Press; 2013.

[34] Telegram Channels, Groups, Bots and Stickers List; n.d. Available from: https://telegramchannels.me.

[35] Lex A, Gehlenborg N, Strobelt H, Vuillemot R, Pfister H. UpSet: Visualization of Intersecting Sets. IEEE Transactions on Visualization and Computer Graphics. 2014;20(12):1983–1992. doi: 10.1109/TVCG.2014.2346248 26356912 PMC4720993

[36] Barron ATJ, Huang J, Spang RL, DeDeo S. Individuals, Institutions, and Innovation in the Debates of the French Revolution. Proceedings of the National Academy of Sciences. 2018;115(18):4607–4612. doi: 10.1073/pnas.1717729115 29666239 PMC5939074

[37] Levshina N. How to do Linguistics with R. Data Exploration and Statistical Analysis. Amsterdam, Philadelphia: John Benjamins Publishing Company; 2015.

[38] fastText Language identification; n.d. Available from: https://fasttext.cc/index.html.

[39] Joulin A, Grave E, Bojanowski P, Mikolov T. Bag of Tricks for Efficient Text Classification. arXiv. 2016; arXiv:1607.01759.

[40] Stuhler O. Who Does What to Whom? Making Text Parsers Work for Sociological Inquiry. Sociological Methods & Research. 2022;51(4):1580–1633. doi: 10.1177/00491241221099551

[41] spaCy: Industrial-strength Natural Language Processing in Python; n.d. Available from: https://spacy.io/.

[42] scikit-learn: machine learning in Python; n.d. Available from: https://scikit-learn.org/stable/index.html.

[43] Gries ST, Divjak D. A Corpus-based Approach to Cognitive Semantic Analysis: Behavioral Profiles. In: Evans V, Pourcel S, editors. New Directions in Cognitive Linguistics. Human Cognitive Processing. John Benjamins Publishing Company; 2009. p. 57–75.

[44] Stano S. The Internet and the Spread of Conspiracy Content. In: Butter M, Knight P, editors. Routledge Handbook of Conspiracy Theories. Conspiracy Theories. New York, NY, USA: Routledge; 2020. p. 483–496.

[45] Leal H. Networked Disinformation and the Lifecycle of Online Conspiracy Theories. In: Butter M, Knight P, editors. Routledge Handbook of Conspiracy Theories. Conspiracy Theories. New York, NY, USA: Routledge; 2020. p. 497–511.

[46] Van Eecke P, Verheyen L, Willaert T, Beuls K. The Candide Model: How Narratives Emerge where Observations Meet Beliefs. In: Proceedings of the The 5th Workshop on Narrative Understanding. Toronto, Canada: Association for Computational Linguistics; 2023. p. 48–57. Available from: https://aclanthology.org/2023.wnu-1.7.

