# Supplementary material for: A computational analysis of Telegram’s narrative affordances
Source: PLoS One. 2023 Nov 14;18(11):e0293508. doi: 10.1371/journal.pone.0293508 (PMC10645302; doi:10.1371/journal.pone.0293508)
Supplement: S1 File — (PDF) [file pone.0293508.s001.pdf]

Supporting information

Abstract

This document provides supporting information with regards to the paper's 1) message classification procedure, 2) retrieved "actants", 3) seed channels used for "snowballing" the datasets, and 4) clustering method.

1 Message classification procedure

This section elaborates on the paper's message classification procedure, which assigns each message to one of six categories ("isolated", "continued", "emerging", "fading", "continued-emerging", "continued-fading") based on the message's scores for "resonance", "novelty" and "transience".

A first two message types can be distinguished when the message's "resonance" score is equal to 0. Firstly, a message's contribution is considered "isolated" if its "novelty" and "transience" have the maximum score (cosine distance) of 1, indicating that there is no overlap between the message's narrative concepts and those in the windows of messages preceding and following it. Secondly, a message's contribution is considered "continued" if its scores for "novelty" and "transience" are equal to each other (yielding a "resonance" of 0), but not equal to 1. This means that in terms of its core narrative concepts, the message is as similar to the ones preceding it as it is to those following it.

Two more categories can be distinguished if the message's "resonance" score is higher than 0. First, a message is considered to be contributing to an "emerging" narrative if has the maximum "novelty" score of 1, and its "transience" score is not equal to 1. This means that the message contributes completely new concepts, which are not completely transient after. Second, a message is considered to be contributing to a "continued-emerging" narrative if its "novelty" score is not equal to 1 and its "transience" score is not equal to 1. This means that the concepts introduced by the message are not completely new and not completely transient, but that these concepts are still more novel than transient. As such, the message can be considered to continue the emergence of a narrative.

Finally, two more categories can be distinguished if a message's "resonance" score is lower than 0. For one thing, if the message has a maximum

“transience” score of 1 and a novelty score that is not equal to 1, this message contributes to a “fading” narrative. In other words, the core narrative concepts it contains have been mentioned in the preceding window of messages, but they are no longer picked up in the ensuing window. For another, if both the message’s “novelty” score and its “transience” score are not equal to 1, we say that the message contributes to a “continued-fading” narrative. This means the narrative concepts figuring in the message are not entirely new, but their “transience” is higher than their “novelty”. This indicates that this message is part of a narrative that is dying out, but, unlike a completely transient “fading” narrative, is still progressing towards its end.

2 Overview of actants

In this section, we provide overviews of the 200 most frequent actants in each of our five datasets, along with a visualisation of the similarities between those lists. As a comprehensive discussion of the narrative contents of the data is beyond the scope of the present paper, we use these overviews to offer a more "distant" reading of dominant themes in each dataset. For each datasets, full lists of actants and their frequencies are made available with the data accompanying this paper. It should be noted that these lists contain references to actants that are highly antagonistic and offensive, including instances of explicitly racist, antisemitic, misogynistic, and anti-LGBTQIA+ discourse.

2.1 Cryptocurrencies

Fig 1 shows a word cloud for the most frequent actants figuring in our dataset pertaining to cryptocurrencies. These comprise financial instruments (e.g. "future"), cryptocurrencies (e.g. "usdc" (USD Coin), "busd" (Binance USD), "bitcoin" (also abbrevatiated as "btc"), "ripple" (also abbreviated as "xrp"), "cardano", "ethereum" (also abbreviated as "eth")), infrastructures for cryptocurrencies (e.g. "blockchain", "wallet"), trading platforms and exchanges for cryptocurrencies (e.g. "binance", "ftx", "coinbase"; "kucoin", "bitfinex"), references to marked actors (e.g. "whale", "investor"), market dynamics ("position", "transaction", "volatility"), and traditional institutions ("government", "bank", "treasury", "regulator").

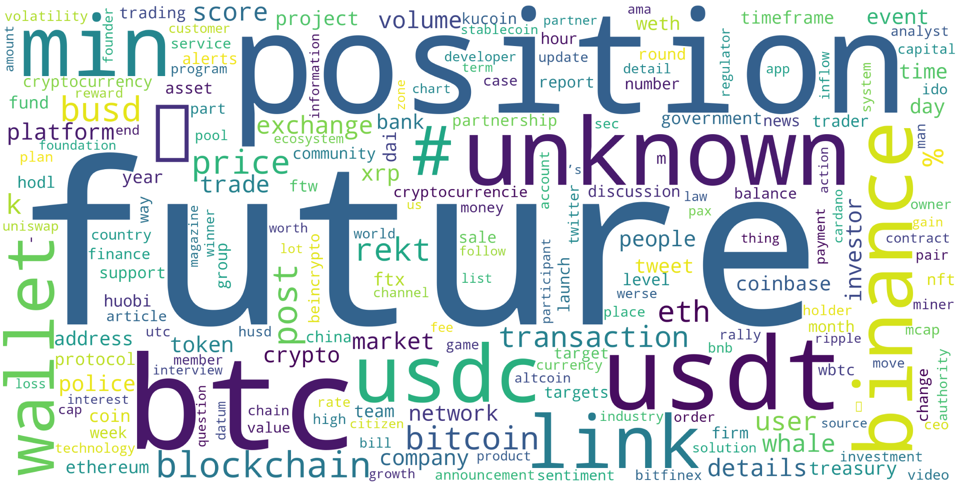

Figure 1: Word cloud of 200 most frequent actants in public Telegram channels "snowballed" from seed channel pertaining to cryptocurrencies

2.2 Stolen elections

Fig 2 shows a word cloud for the most frequent actants figuring in our dataset pertaining to the "stolen elections" conspiracy theory. This concerns (former) U.S. presidents (e.g. "trump", "biden"), political actors associated with the Republican Party (e.g. "flynn", "desantis"), political actors associated with the Democratic Party ("obama", "clinton", "pelosi"), elections and actors in electoral processes (e.g. "election", "ballot", "vote"), administrative institutions (e.g. "state", "government", "administration", "state"), geopolitical actors (e.g. "russia", "china", "ukraine", "putin", "israel"), actors in law enforcement (e.g. "court", "investigation", "fbi", "police", "law", "lawyer", "lawsuit", "judge", "doj"), religious actors (e.g. "god"), nationalist actors (e.g. "patriot"), actors connected with vaccines (e.g. "virus", "vaccine", "fauci"), and actors central to the QAnon conspiracy theory (e.g. "q").

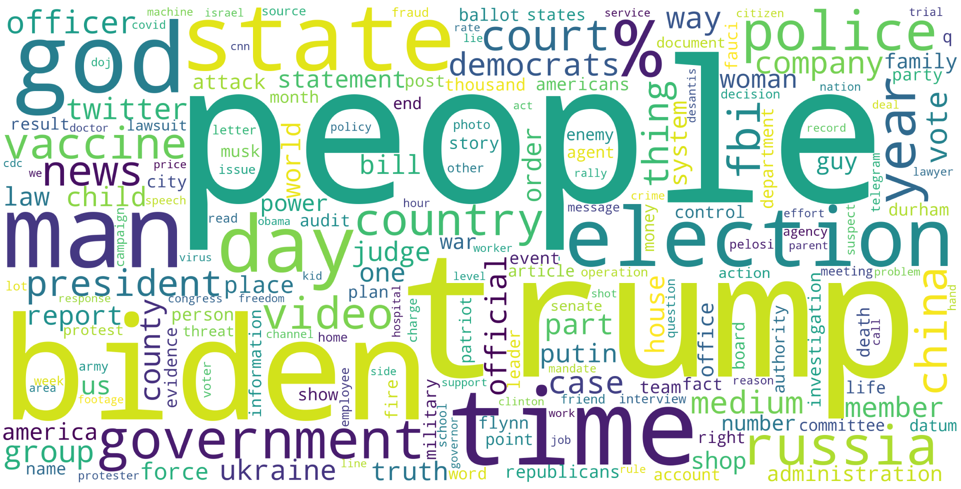

Figure 2: Word cloud of 200 most frequent actants in public Telegram channels "snowballed" from seed channel pertaining to the pro-Trump "stolen elections" conspiracy theory

2.3 Far-right counterculture

Fig 3 shows a word cloud with the most frequent actants figuring in the Telegram dataset pertaining to far-right counterculture. This concerns actants related to ethnoreligious groups (e.g. "jews"), historical actors ("hitler"), religious actors (e.g. "christ", "god", "jesus", "prayer", "church"), institutions (e.g. "state", "government"), actants pertaining to incidents and protests (e.g. "shooting", "shooting", "incident", "protestor", "protest", "attack"), actants from law enforcement (e.g. "police", "officer", "fbi"), and actants associated with vaccines (e.g. "vaccine").

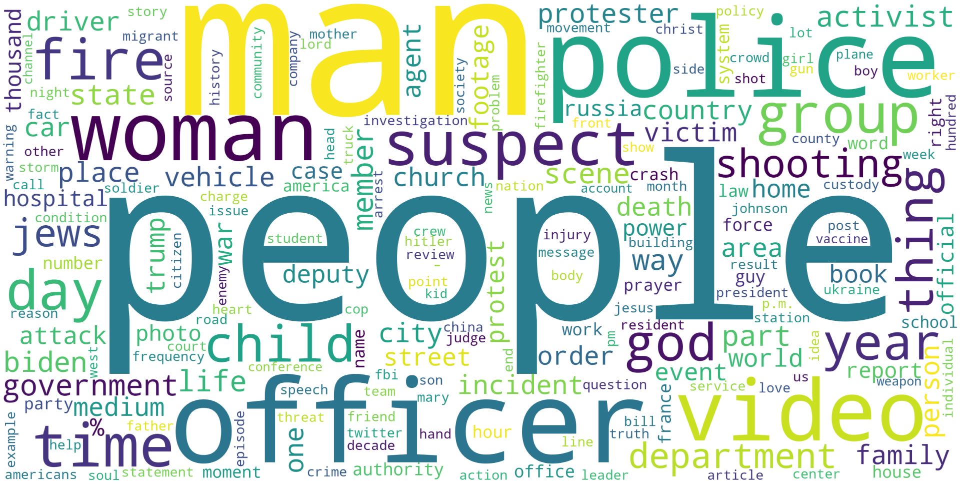

Figure 3: Word cloud of 200 most frequent actants in public Telegram channels "snowballed" from seed channel pertaining to far-right counterculture

2.4 The “Great Reset”

Fig 4 shows a word cloud with the most frequent actants figuring in our dataset of channels “snowballed” from a seed channel associated with the “Great Reset” conspiracy theory. This concerns (former) U.S. presidents (e.g. “trump”, “biden”), geopolitical actors (e.g. “china”, “russia”, “ukraine”, “putin”, “israel”), actants associated with covid and vaccines (e.g. “vaccine”, “covid”, “fauci”, “virus”), administrative institutions (e.g. “state”, “government”, “administration”), actors from law enforcement (e.g. “officer”, “police”), economic actors (e.g. “company”, “money”, “stock”, “market”, “inflation”), and religious actors (e.g. “god”). It should be noted that actants central to conspiracy actors (e.g. “q”) figure among the 300 most frequent actants in the dataset.

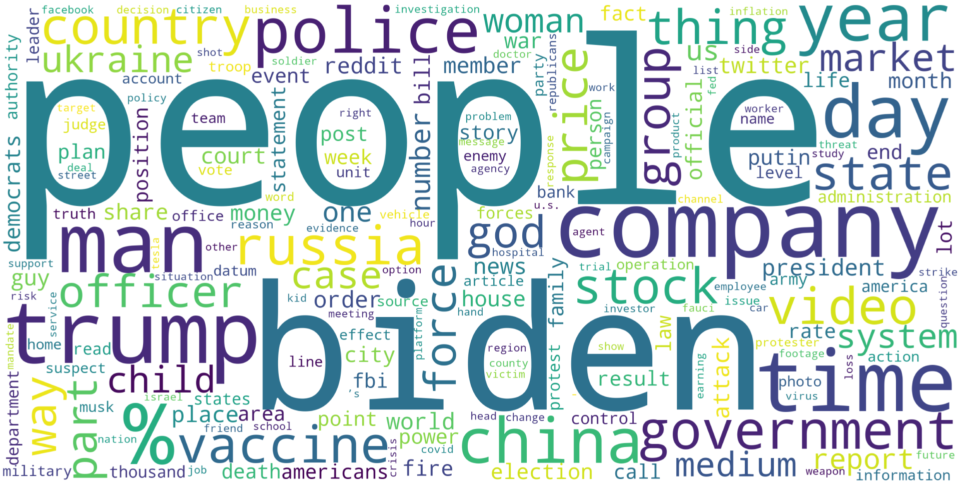

Figure 4: Word cloud of 200 most frequent actants in public Telegram channels “snowballed” from seed channel pertaining to the “Great Reset” conspiracy theory

2.5 Ukraine war

Fig 5 shows a word cloud with the most frequent actants figuring in our dataset of channels "snowballed" from a seed channel pertaining to the war in Ukraine. This comprises geopolitical actants (e.g. "europe", "eu", "germany", "nato", "russia", "ukraine", "china", "azerbaijan", "israel", "poland", "moscow"), world leaders (e.g. "zelensky", "biden", "putin" , "trump", "bolsonaro"), military actants ("troop", "military", "forces", "army", "unit", "soldier", "operation", "offensive", "missile", "rocket", "attack", "battle", "helicopter"), as well as other actants associated with armed conflict (e.g. "terrorist", "mercenary").

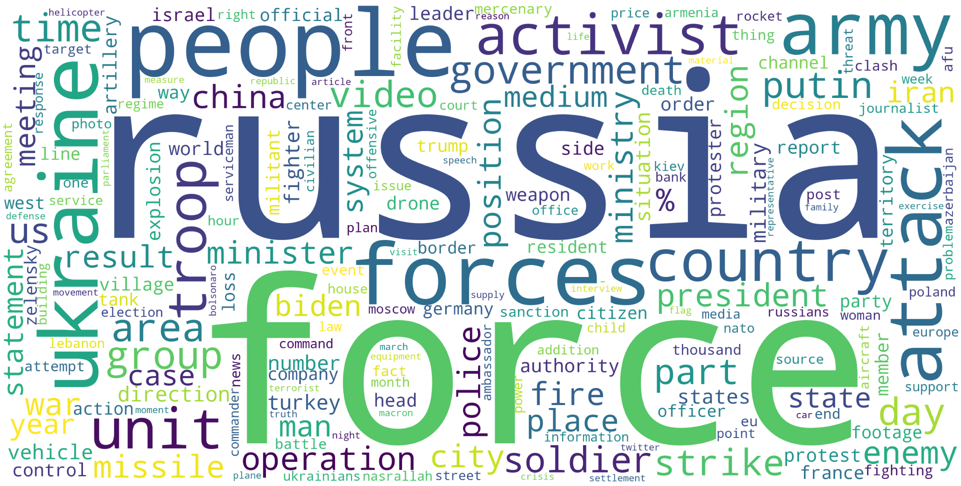

Figure 5: Word cloud of 200 most frequent actants in public Telegram channels "snowballed" from seed channel pertaining to the war in Ukraine

2.6 Similarities between lists of actants

Fig 6 illustrates the similarities between lists of the top 200 most frequent actants for each of our five datasets. Similarity is indicated by means of the Jaccard coefficient, which corresponds with the the size of the intersection of two sets of actants divided by the size of the union of these sets. The heatmap indicates that the actant lists pertaining to the stolen elections conspiracy theory dataset and the "Great Reset" conspiracy theory are most similar. The far-right counterculture dataset, in terms of the lists of the 200 most frequent actants, is most dissimilar to these two datasets.

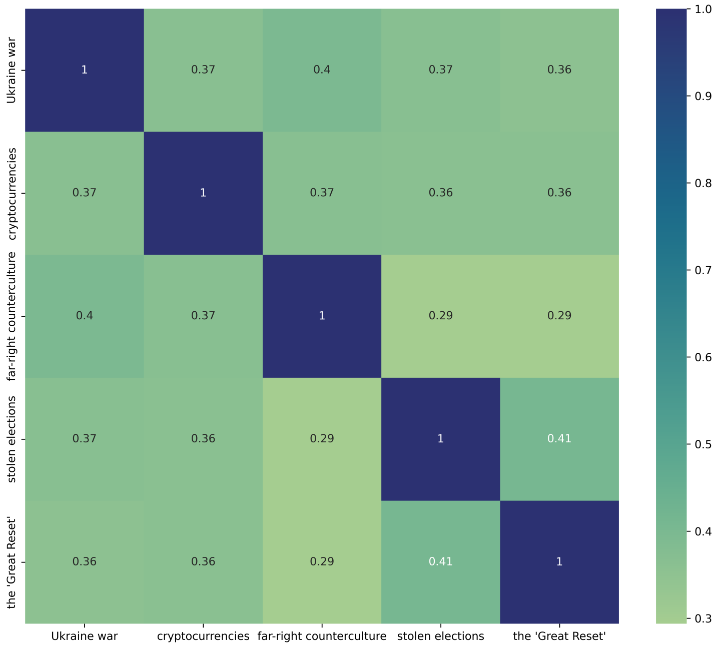

Figure 6: Heatmap of Jaccard similarities between lists of 200 most frequent actants for each of the five datasets.

3 Seed channels

All of the data for this paper have been collected in the public interest from publicly available sources. No personal data were collected or retained in this process. Still, following best practices outlined in [1], we have chosen to pseudonymize the names of public Telegram channels discussed in this paper. The central motivation behind this choice is that while public Telegram channels can be previewed from a browser even by people who do not have a Telegram account, it cannot be assumed that their contents are openly available for distribution in a different (in the present case, scientific) context. The names of the public Telegram channels that form the seeds of our snowballing efforts have thus been replaced with fictional names. This renaming was done in such a way that the pseudonyms offer an indication of the channel's theme without unnecessarily exposing them. The list of seed channels and their creation dates is presented in Table 1.

**Dataset**	**Channel name**	**Creation date**
Cryptocurrencies	coin_notifications	2019
Stolen elections	TakenByStorm17	2021
Far-right counterculture	pilledliteraturereadings	2021
The "Great Reset"	GreatResetChronicle	2021
Ukraine war	intelligence_for_victory	2020

Table 1: Overview of seed channels. Names of channels have been pseudonymized.

4 Clustering method

In our hierarchical clustering analysis, we make use of the Ward variance minimization algorithm to calculate the distances between clusters. The Ward algorithm was selected on the grounds that it produces compact clusters [2, p. 311]. In this section, we provide further validation for this choice by comparing the clusters under discussion with those retrieved through other linkage methods. We thereby focus on the clusters that are obtained when applying different methods to our aggregated dataset of Telegram channels. The selection of linkage methods presented here is based on [2, p. 310-311], with implementations following those of the SciPy python library [3]. We specifically contrast the outcomes of the Ward method in Fig 7 with the "single" (Nearest Point Algorithm) method in Fig 8, with the "complete" (Farthest Point Algorithm) method in Fig 9, and with the "average" (UPGMA algorithm) method in Fig 10.

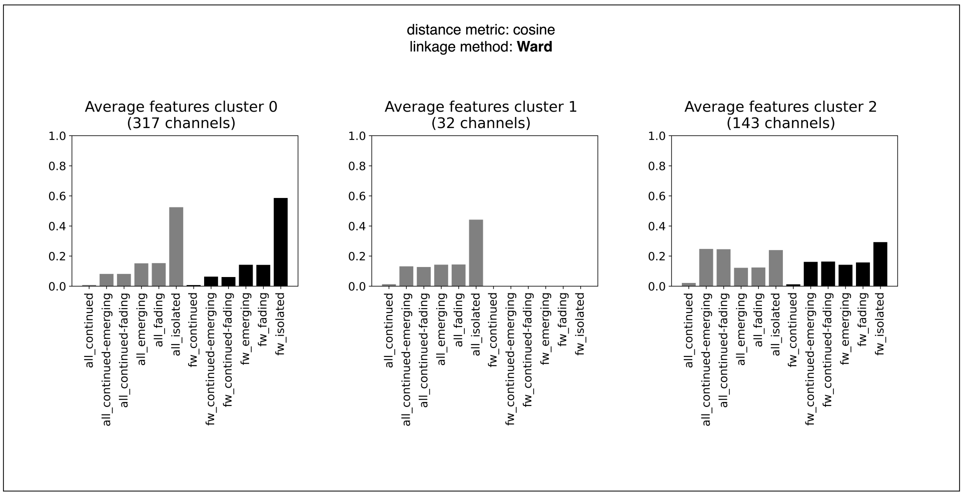

Figure 7: Narrative profiles for aggregated analysis of all 492 Telegram channels in the dataset. Agglomerative hierarchical clustering of BP vectors based on cosine distance and Ward linkage method.

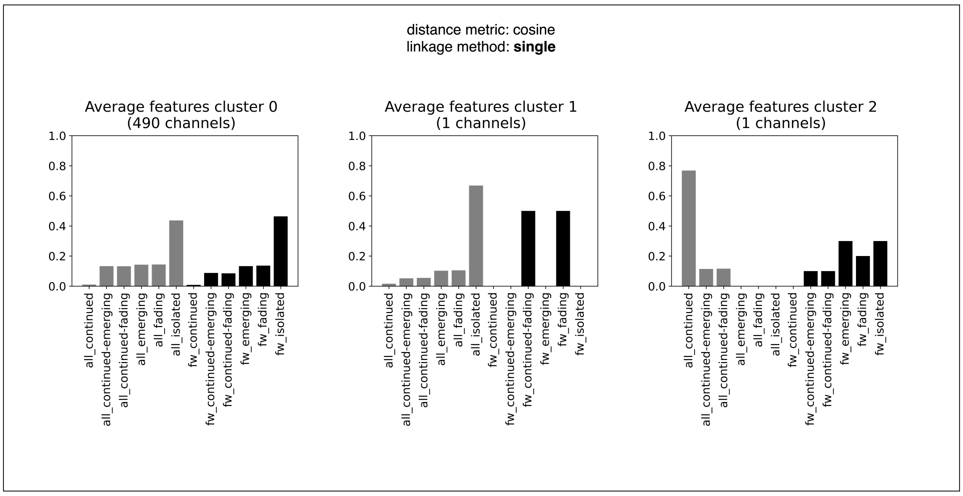

Figure 8: Narrative profiles for aggregated analysis of all 492 Telegram channels in the dataset. Agglomerative hierarchical clustering of BP vectors based on cosine distance and "single" linkage method.

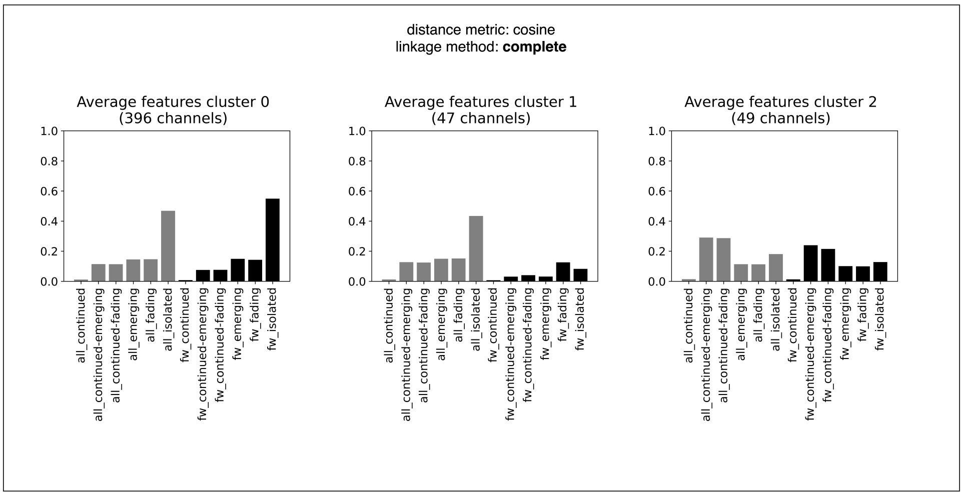

Figure 9: Narrative profiles for aggregated analysis of all 492 Telegram channels in the dataset. Agglomerative hierarchical clustering of BP vectors based on cosine distance and "complete" linkage method.

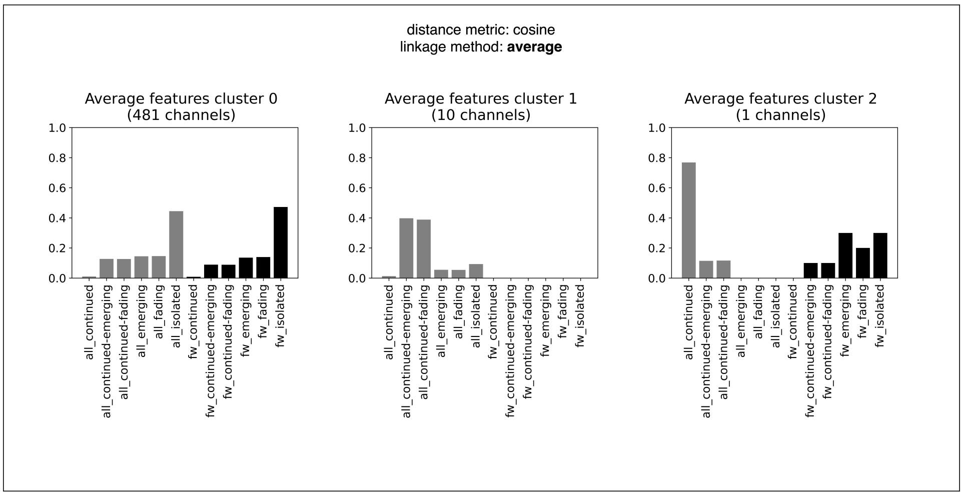

Figure 10: Narrative profiles for aggregated analysis of all 492 Telegram channels in the dataset. Agglomerative hierarchical clustering of BP vectors based on cosine distance and "average" linkage method.

References

[1] Willaert T, Peeters S, Seijbel J, Van Raemdonck N. Disinformation Networks: A Quali-Quantitative Investigation of Antagonistic Dutch-speaking Telegram Channels. First Monday. 2022;27(5). doi:10.5210/fm.v27i5.12533.

[2] Levshina N. How to do Linguistics with R. Data Exploration and Statistical Analysis. Amsterdam, Philadelphia: John Benjamins Publishing Company; 2015.

[3] SciPy documentation;. Available from: `https://docs.scipy.org/doc/scipy/`.
